# LITH: A Lightweight Behavioral Anomaly-to-Hunt-Lead Framework for IoT Sensor Ecosystems

**DOI:** 10.3390/s26206618

**Published:** 2026-10-19

**Authors:** Fatmah Bamashmoos, Enas Khairullah

**Affiliations:** Faculty of Computing & Information Technology, King Abdulaziz University, Jeddah 21589, Saudi Arabia; ekhairallah@kau.edu.sa

**Keywords:** Internet of Things, IoT security, anomaly detection, threat hunting, sensor networks, resource-constrained devices, edge computing, isolation forest

## Abstract

Internet of Things (IoT) sensor ecosystems increasingly support critical services, yet many endpoints cannot host conventional security stacks because of limited processing and memory resources. This paper presents the Lightweight IoT Threat Hunter (LITH), a hybrid endpoint-edge-central anomaly-to-hunt-lead framework that converts compact behavioral telemetry into calibrated, ranked investigation leads for downstream threat-hunting workflows while keeping automated anomaly detection separate from analyst confirmation. The framework was evaluated using a controlled multidevice telemetry benchmark across 10 Monte Carlo runs, with benign-only model fitting and validation-based anomaly-score calibration. At the common 99th-percentile operating point, One-Class Support Vector Machine (SVM) achieved the highest mean thresholded F1-score, although its difference from the autoencoder was not statistically significant after Holm correction, while Isolation Forest achieved the strongest mean Area Under the Receiver Operating Characteristic Curve (ROC-AUC) and average precision. Network-only telemetry retained performance close to the combined network-and-system feature set, while system-only telemetry was substantially weaker for the evaluated attack scenarios. Robustness and operating-point analyses showed that performance degrades under weaker attacks and increased benign variability and that higher sensitivity to subtle exfiltration behavior requires accepting a larger benign investigation workload. Beyond anomaly classification, LITH converts high-scoring observations into device-class-specific ranked hunt leads with behavioral indicators intended to support analyst triage rather than autonomous threat attribution. A limited three-feature external stress test on the Canadian Institute for Cybersecurity’s (CICIoT2023) dataset produced markedly different detector behavior: Max-Z achieved an F1-score of 0.824 and ROC-AUC of 0.835, while Isolation Forest did not transfer reliably at the fixed 99th-percentile operating point. This class-dependent result shows that the detector ranking observed in the controlled benchmark does not generalize uniformly. The results support proof-of-concept feasibility while emphasizing that broader device-aware external validation and physical endpoint and edge measurements remain necessary before claims of generalization or deployment readiness can be made.

## 1. Introduction

Internet of Things (IoT) technologies now underpin smart buildings, environmental monitoring, healthcare sensing, industrial automation, and urban infrastructure. These services rely on heterogeneous sensors, cameras, actuators, and gateways that are frequently inexpensive, long-lived, and resource constrained. Their operational value therefore coexists with persistent security weaknesses, including weak credentials, unpatched firmware, exposed services, and limited capacity to run endpoint-security software [1,2].

Signature-based intrusion detection remains effective when reliable indicators of known attacks are available, while specification-based controls can enforce expected behavior for tightly defined device classes [3,4]. However, both approaches face limitations in dynamic IoT environments. A previously unseen compromise may first appear as a behavioral deviation—for example, a sensor increasing its transmission rate, a camera contacting an unfamiliar destination, or an actuator scanning neighboring devices—before a recognizable signature or rule violation is available. Behavioral anomaly detection therefore provides an important complementary layer by modeling expected device behavior and flagging departures that warrant further investigation [5,6,7,8,9].

Recent IoT-security research increasingly emphasizes lightweight detection, edge processing, and deployment-aware architectures. Edge security can externalize expensive functions from constrained endpoints [10,11], flow-based and anomaly-based methods can reduce dependence on attack signatures [12,13,14], and recent lightweight Intrusion Detection System (IDS) designs explicitly optimize feature selection, novelty detection, memory use, or energy efficiency [14,15,16,17,18]. These developments improve deployability, but much of the literature remains detector-centric: the anomaly score or classifier output is treated as the endpoint of the security workflow.

Threat hunting addresses a related but broader operational objective. In prior Industrial Internet of Things (IIoT) research, threat-hunting models have used deep autoencoders, recurrent networks, federated learning, or ensembles to identify suspicious activity [19,20]. Such work demonstrates the value of proactive behavioral analysis, but it also highlights a gap for simpler architectures that fit heterogeneous, resource-constrained sensor ecosystems. In this paper, threat hunting does not mean that an anomaly score proves compromise. Instead, the score is used to prioritize a device and provide contextual evidence for Security Information and Event Management (SIEM) or analyst investigation. This distinction is central to the design of LITH.

The Lightweight IoT Threat Hunter (LITH) is therefore positioned as a system-level anomaly-to-hunt-lead framework that connects minimal telemetry, edge preprocessing, lightweight unsupervised modeling, and ranked investigation leads. The term “Threat Hunter” in LITH denotes this hunt-support role: the framework produces prioritized, contextualized leads for downstream analyst- or SIEM-supported investigation rather than autonomously performing the complete threat-hunting lifecycle. LITH does not claim threat confirmation, malware attribution, autonomous response, or a new anomaly-detection algorithm.

The main contributions are as follows:

- A hybrid endpoint-edge-central architecture that minimizes endpoint processing while preserving sufficient behavioral context for security analysis.
- A compact eight-feature telemetry design combining network and, where available, system indicators for heterogeneous sensor-device profiling.
- A lightweight analytics layer based on Isolation Forest, One-Class SVM, and a shallow autoencoder, together with a simple Max-Z statistical baseline for context.
- An explicit anomaly-to-hunt-lead workflow that transforms high-scoring deviations into ranked, contextualized investigation leads for downstream analyst- or SIEM-supported threat hunting, while keeping anomaly detection separate from threat confirmation and analyst validation.
- A reproducible proof-of-concept evaluation using device-class-specific benign-only temporal training, 10 Monte Carlo runs, attack-specific analysis, feature ablation, matched-seed significance testing, analytics-side footprint measurements, and an attack-strength/benign-variability sensitivity study.

## 2. Related Work and Research Gap

### 2.1. Lightweight Intrusion and Anomaly Detection in IoT

IoT intrusion-detection research spans signature-based, specification-based, statistical, machine learning, and deep learning approaches [3,18,21]. The deployment problem is especially important for constrained endpoints. Gyamfi and Jurcut [10] reviewed the use of multiaccess edge computing to offload IDS functions, while Canavese et al. [11] demonstrated the broader principle of externalizing security functions to an edge gateway for resource-limited devices. Zachos et al. [14] implemented anomaly-based intrusion detection for resource-constrained Internet of Medical Things (IoMT) devices using a lightweight monitoring component and a remote detection engine, and Almalawi [15] reduced IDS cost through data condensation and stable-feature selection. These studies reinforce the need to evaluate security mechanisms not only by detection metrics but also by deployment cost. Device-type identification can further support class-specific security policies and behavioral baselines in heterogeneous IoT deployments [22].

### 2.2. Behavioral Anomaly Detection and Edge Analytics

Behavioral anomaly detection is attractive when the defender cannot enumerate all malicious patterns. Meidan et al. [5] proposed N-BaIoT, a network-based method for detecting IoT botnet attacks using deep autoencoders, and subsequent surveys have cataloged a broad range of machine learning and deep learning methods while identifying persistent concerns around false positives, dataset realism, computational cost, and generalization [6,7,13,18]. Mutambik [12] proposed a flow-based IoT anomaly detector that learns from benign traffic, while Reis [16] demonstrated a sensor-network anomaly pipeline emphasizing edge processing and efficiency. These works motivate LITH’s emphasis on compact telemetry and centralized modeling, but they do not by themselves define an analyst-facing threat-hunting process. Interpretability is also increasingly considered in IoT intrusion detection because an analyst must understand why a model has flagged an observation. Sharma et al. [23] combined deep-learning-based IoT intrusion detection with local interpretable model-agnostic explanations (LIME) and SHapley Additive exPlanations (SHAP) explanations to identify the features contributing to individual predictions. This work illustrates the value of exposing behavioral evidence alongside a detection result. LITH pursues a related analyst-facing objective but does not apply a post hoc explainability model; instead, it attaches the largest standardized behavioral deviations directly to each ranked investigation lead.

The scope of IoT anomaly detection can also extend beyond the behavior of an individual device. Recent surveys describe anomalies arising across multiple layers of the IoT architecture [24], while complementary work has addressed semantic interference and conflicts among trigger–action rules and industrial IoT control policies. For example, the Scene Interaction Graphs for Interference Detection (SIGFRID) approach targets platform-agnostic interference detection among IoT automation rules [25], while formal rule-conflict approaches model inconsistent or interacting control policies in Industrial IoT systems [26]. These approaches address cross-component semantics that are outside the present device-class-level LITH telemetry model.

### 2.3. Threat Hunting in IoT and IIoT

Threat-hunting research in IoT has often been coupled with deep or distributed learning. Yazdinejad et al. [19] combined long short-term memory (LSTM) and autoencoder components for IIoT threat hunting, while Jahromi et al. [20] used federated representation learning and ensemble decision making to support privacy-preserving cyber-threat hunting. These contributions target strong detection performance and collaborative learning. LITH addresses a different point in the design space: it favors minimal behavioral telemetry and lightweight unsupervised scoring, then treats the output as an investigation lead rather than an autonomous threat verdict.

A closely related line of research concerns security-alert triage and prioritization. Wang et al. [27] proposed AlertPro, a context-aware alert-prioritization framework designed to reduce alert fatigue by ranking alerts for analyst attention during security operations center (SOC) triage and validation. This operational perspective is relevant to LITH because both approaches seek to direct limited analyst effort toward higher-priority observations. However, LITH ranks device-class-specific anomaly leads using calibrated behavioral evidence rather than prioritizing correlated multistep SOC alerts, and it leaves threat confirmation to downstream analyst or SIEM investigation. Table 1 summarizes the qualitative positioning of LITH relative to representative related studies.

### 2.4. Research Gap

The literature provides strong evidence for anomaly detection, edge offloading, and learning-based threat hunting, but fewer studies connect all three under a deliberately lightweight operational design for heterogeneous sensor endpoints. LITH addresses this gap by separating sensing/collection, edge reduction, centralized behavioral scoring, and investigation prioritization. The framework is intentionally conservative: it aims to improve the quality and timeliness of investigation leads, not to replace the analyst or claim autonomous attribution.

## 3. Materials and Methods

### 3.1. Design Objectives and Threat Model

LITH is designed around four objectives: (i) minimize processing on constrained endpoints, (ii) use compact telemetry that remains useful across heterogeneous device types, (iii) detect behavior that deviates from an established device profile without requiring attack signatures in the scoring model, and (iv) convert anomalies into prioritized investigation leads rather than final security decisions.

The threat model assumes that an attacker can compromise IoT devices through weak credentials, software vulnerabilities, exposed services, or lateral movement. After compromise, the attacker may enroll a device in distributed denial-of-service (DDoS) activity, exfiltrate data to an unexpected destination, or scan neighboring services. The edge collectors and central analytics engine are assumed to remain trusted, and telemetry is assumed not to be modified before collection. Hardware-level compromise, compromise of the analytics infrastructure, and attacks that do not materially affect the selected telemetry are outside the scope of the present proof of concept. Encrypted payload inspection is not required because LITH operates on behavioral metadata; however, attacks that preserve normal metadata patterns may evade detection. The present implementation models behavioral deviations at the device-class level and does not explicitly model semantic dependencies, causal interactions, or conflicts among multiple devices and automation rules.

### 3.2. LITH Architecture and Threat-Hunting Workflow

**Figure 1:**
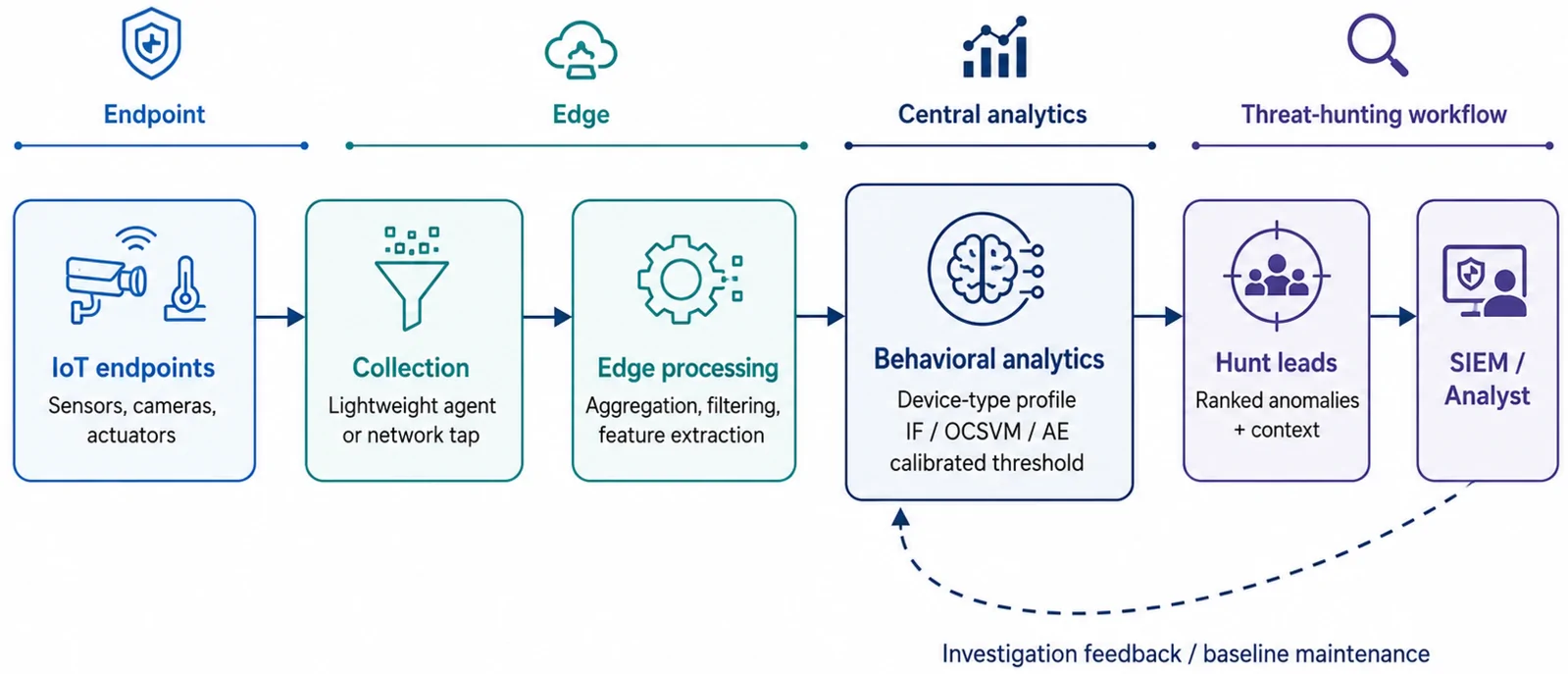
LITH hybrid architecture. Lightweight collection and edge preprocessing feed device-class-specific behavioral analytics; high-scoring deviations are converted into ranked hunt leads for SIEM or analyst investigation. The dashed investigation-feedback/baseline-maintenance loop represents a future extension and is not implemented or evaluated in the present proof of concept.

Figure 1 shows the LITH architecture. Endpoint monitoring is intentionally narrow: a lightweight agent, gateway observer, or network tap obtains compact behavioral indicators. The edge layer aggregates observations, applies basic filtering, and extracts the configured features. Central analytics maintains a separate behavioral profile for each device class and applies an unsupervised anomaly model. Each raw anomaly score is calibrated against the benign validation-score distribution for the corresponding device class. In the current implementation, hunt leads are ranked primarily by the calibrated anomaly percentile, with the raw anomaly score used as a secondary ordering key for tied observations within the same detector and device class; no secondary learned risk-prioritization model is applied. An event above the decision threshold is therefore treated as an investigation lead, not as a confirmed compromise. The lead contains device identity, anomaly percentile, changed features, and available network context for SIEM or analyst validation. The feedback path shown in Figure 1 is conceptual future work; the current implementation does not automatically incorporate analyst decisions into baseline maintenance or model updating.

### 3.3. Behavioral Telemetry

LITH uses an eight-dimensional feature vector selected to balance behavioral coverage and monitoring cost (Table 2). Five features are network-derived and can be collected by a gateway or network tap. Three are optional system-level features when host telemetry is available. The protocol feature is represented as the fraction of packets in the observation that use the User Datagram Protocol (UDP) fraction of packets in the observation. To preserve packet-level physical consistency, the total packet count is first represented as a positive integer, the implied number of UDP packets is rounded to an integer between zero and the total packet count, and the UDP ratio is then reconstructed as the integer UDP-packet count divided by the total packet count. Packet count, unique-destination count, and process count are discrete integer-valued variables in the generator, and the unique-destination count is constrained not to exceed the corresponding total packet count. Packet-size variability is defined explicitly as the standard deviation of packet size in bytes. Because the controlled benchmark operates directly at the feature-vector level, it evaluates behavioral separability rather than the effect of a particular wall-clock aggregation-window duration.

### 3.4. Controlled Telemetry Benchmark

A controlled telemetry benchmark was implemented to evaluate LITH under a fully reproducible benign-only training and validation protocol. The benchmark instantiates the three device behaviors described by LITH at the feature-vector level. In each baseline Monte Carlo run, 50,000 benign observations are generated: 16,667 environmental-sensor observations, 16,667 smart-camera observations, and 16,666 actuator observations. A mild sinusoidal drift with 2.5% amplitude is applied to selected normal-behavior variables before the temporal split. For each benign device-class sequence, the sinusoid completes one full cycle over the complete generated sequence; consequently, the 60%/20%/20% temporal training, validation, and test partitions correspond to the first 60%, next 20%, and final 20% of that cycle, respectively. Device-class phase offsets of 0.00, 0.17, and 0.34 cycles are used for the sensor, camera, and actuator profiles.

Attack observations are generated independently as test-only samples from benign-like bases before the corresponding attack perturbations are applied. Each attack set also spans one complete sinusoidal drift cycle, with phase offsets of 0.15 for DDoS, 0.25 for exfiltration, and 0.35 for reconnaissance. The attack observations are therefore not temporally aligned as either the same or the opposite phase of the benign test segment; the phase offsets specify only the starting position of a complete cycle and do not change its marginal sinusoidal drift distribution. Stochastic variation and drift amplitude are increased systematically in the robustness experiment described below.

The environmental-sensor profile represents low-rate behavior resembling Message Queuing Telemetry Transport (MQTT) traffic, with packet activity centered around approximately seven packets per observation, low UDP ratio, and low CPU and memory use. The smart-camera profile represents high-rate behavior resembling Real-time Transport Protocol (RTP)traffic, with integer packet activity centered around approximately 950 packets per observation, mean packet size around 1080 bytes, high UDP ratio, and higher resource use. The actuator profile represents low-rate command/status traffic with packet activity centered around approximately eleven packets per observation and low resource use. All count-valued variables are generated from discrete distributions or rounded to integer values and then bounded to physically valid ranges. In addition, the number of unique destinations is constrained not to exceed the total packet count, and the UDP ratio is reconstructed from an integer UDP-packet count divided by the corresponding integer total packet count. A physical-consistency audit was applied to the generated benchmark to verify these constraints. Exact distributions, clipping ranges, random-generation code, and the physical-consistency audit are provided in the Supplementary Materials package.

**Figure 2:**
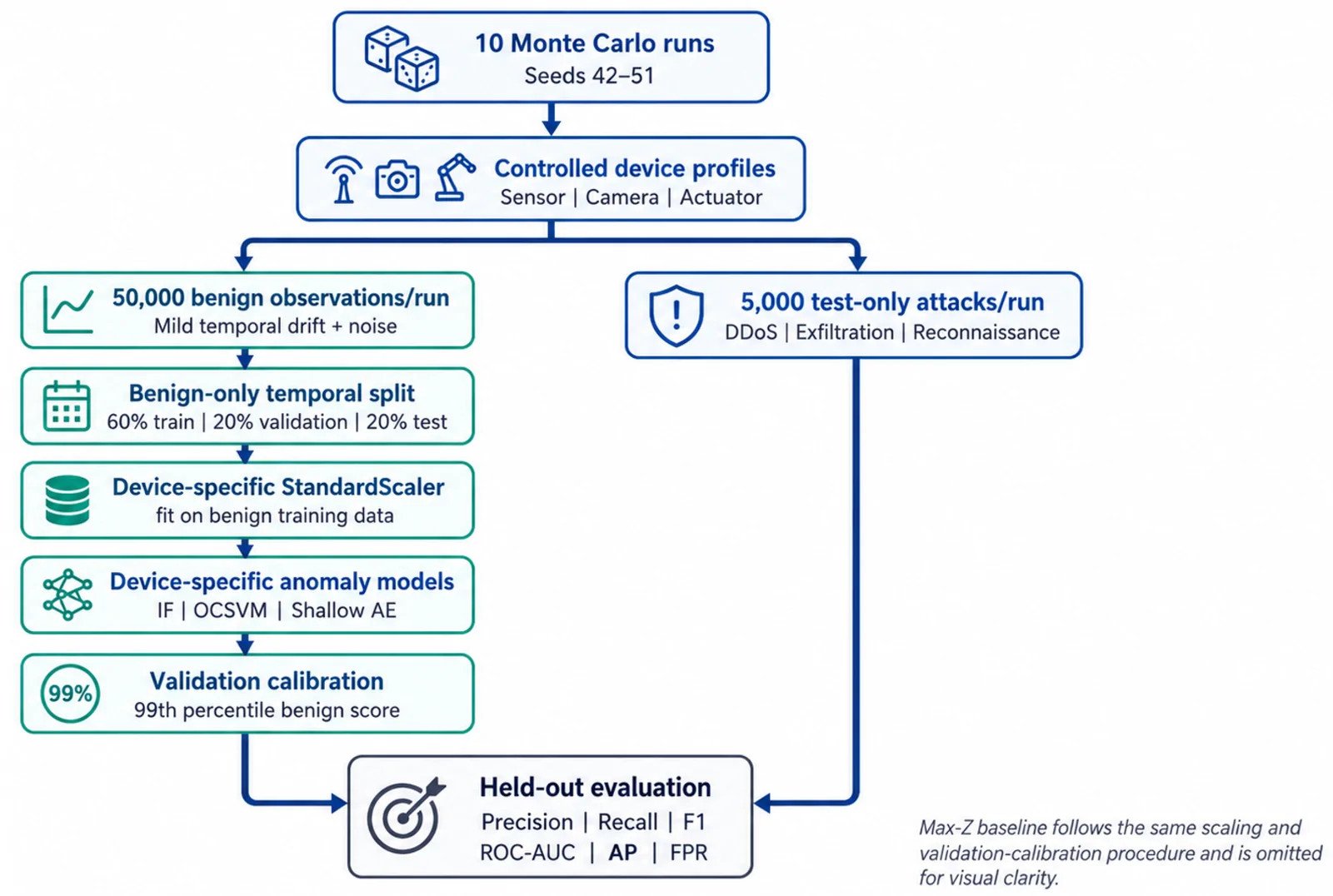
Controlled telemetry benchmark and baseline evaluation protocol. The experiment is repeated across 10 Monte Carlo runs using random seeds 42–51. For each run, benign observations from environmental-sensor, smart-camera, and actuator profiles are divided temporally into training, validation, and test partitions, while attack observations are excluded from model fitting and threshold calibration and appear only in the held-out test set. Device-class-specific standardization and anomaly models are fitted using benign training data, and anomaly scores are calibrated against the corresponding benign validation distribution using a 99th-percentile decision threshold. The Max-Z statistical baseline is evaluated using the same scaling and validation-calibration procedure but is omitted from the diagram for visual clarity. Green boxes identify benign-data preparation, detector fitting, and calibration. Blue boxes identify the experimental setup and test-only attack branch, while the gray box denotes held-out evaluation. Colors distinguish workflow stages and do not encode numerical values.

A further 5000 attack observations are generated per run and are used only in the held-out test set: 1667 DDoS observations, 1667 exfiltration observations, and 1666 reconnaissance observations. DDoS modifies the sensor profile by sharply increasing packet rate, UDP ratio, destination behavior, packet-size standard deviation, and CPU demand. Exfiltration modifies the camera profile more subtly by adding an unfamiliar Transmission Control Protocol (TCP) transfer, increasing destination count and packet-size standard deviation while reducing UDP ratio and moderately increasing CPU and memory use. Reconnaissance modifies the actuator profile by increasing connection attempts and unique destinations while producing smaller, scan-like traffic. Additional methodological details, physical-consistency checks, and supporting analyses are provided in the Supplementary Materials. The experimental code and reproducibility outputs are also available through the public GitHub repository identified in the Data Availability Statement. Figure 2 summarizes the baseline evaluation flow.

### 3.5. Preprocessing and Behavioral Models

A separate StandardScaler is fitted to the benign training partition of each device class. The same scaler is then applied to that device class’s validation and test observations. For computational parity across the learned models, 2000 benign training observations are sampled without replacement per device class, giving 6000 benign training observations per model in each Monte Carlo run. This fixed fitting budget was used to keep the learned detectors on a common training-sample basis and to limit the computational growth of the kernel-based One-Class SVM. To assess whether this subsampling materially affects the conclusions, a separate training-size sensitivity analysis was performed. In that analysis, Isolation Forest and One-Class SVM were fitted using all available benign training observations (9999–10,000 per device class), while the autoencoder retained the same disjoint 1500-observation early-stopping holdout and used all remaining benign training observations (8499–8500 per device class). All other detector hyperparameters, calibration procedures, and test data were kept unchanged. Attack labels are not used for feature scaling, model fitting, early stopping, or threshold calibration.

Isolation Forest uses 100 estimators, max_samples = 256, max_features = 1.0, and contamination=’auto’. The built-in contamination offset is not used as the reported decision rule; only the continuous anomaly score is retained and subsequently calibrated against benign validation scores. For subsample size ψ, the path-length normalization factor is

(1) $$c\left(\psi \right)=2H\left(\psi -1\right)-\frac{2(\psi -1)}{\psi },$$

and the anomaly score is

(2) $$s\left(x,\psi \right)=2^{-\frac{E[h(x)]}{c(\psi )}},$$

where E[h(x)] is the average path length across the isolation trees and $H\left(k\right)=\sum_{j=1}^{k}\frac{1}{j}$ denotes the *k*th harmonic number. For sufficiently large *k*, it can be approximated as H(k)≈ln(k)+γ, where γ≈0.5772 is the Euler-Mascheroni constant [28]. In the implementation, scikit-learn’s IsolationForest.score_samples() output is sign-inverted,

(3) $$r_{\mathrm{IF}}\left(x\right)=-\mathrm{score}\_\mathrm{samples}\left(x\right),$$

Because the library output assigns lower values to more abnormal observations. The sign inversion therefore ensures that larger values consistently indicate greater anomaly evidence before calibration.

The use of 100 trees was specified a priori as a moderate ensemble size intended to balance score stability and model footprint; it was not selected using attack labels. To assess sensitivity to this choice, a post hoc analysis additionally evaluated 50 and 200 trees while keeping all other Isolation Forest settings, benign-only fitting, validation calibration, and test data unchanged.

One-Class SVM uses a radial basis function (RBF) kernel with ν=0.1, γ=scale, tolerance 0.001, and a 1000 MB kernel cache. For One-Class SVM, the implementation uses the negative signed distance to the separating hyperplane,

(4) $$r_{\mathrm{OCSVM}}\left(x\right)=-\mathrm{decision}\_\mathrm{function}\left(x\right).$$

The native decision function is positive for inliers and negative for outliers; negating it again establishes the common convention that larger raw scores correspond to greater anomaly evidence. The shallow autoencoder follows a fully connected architecture with layer widths 8→6→4→3→4→6→8, rectified linear unit (ReLU) hidden activations, and a linear output. It is trained with Adam (η=0.001) and mean-squared reconstruction loss for at most 25 epochs, batch size 256, with early-stopping patience of 4 epochs. For the autoencoder, a separate holdout of 1500 benign observations per device class is drawn from the training partition after excluding the 2000 observations used for model fitting. This disjoint training-partition holdout is used only for early stopping, while the full benign validation partition is reserved exclusively for anomaly-score calibration. The autoencoder anomaly score is the mean squared reconstruction error across the standardized input features. For an observation with standardized feature vector z and reconstruction $\hat{z}$,

(5) $$r_{\mathrm{AE}}\left(z\right)=\frac{1}{d}\sum_{i=1}^{d}{\left(z_{i}-{\hat{z}}_{i}\right)}^{2},$$

where d=8 in the combined-feature configuration. Larger reconstruction errors indicate greater deviation from the learned benign representation. Across all 30 autoencoder fits (3 device classes across 10 Monte Carlo seeds), training reached the maximum of 25 epochs; the early-stopping criterion was therefore not triggered before the predefined epoch limit.

The three-unit bottleneck was likewise specified a priori rather than selected using attack labels. With an eight-dimensional input, it provides a compact latent representation while retaining a shallow symmetric architecture. A post hoc sensitivity analysis varied only the bottleneck width over 2, 3, and 4 units, while preserving the same optimizer, learning rate, training budget, disjoint benign early-stopping holdout, calibration procedure, and test set.

A simple statistical comparator, denoted Max-Z, is included to test whether the controlled benchmark can be solved equally well without a learned anomaly model. After device-class-specific standardization, its raw anomaly score is

(6) $$r_{\mathrm{Max}-Z}\left(z\right)=\max_{i}\left|z_{i}\right|,$$

where $z_{i}$ is the standardized value of feature *i* obtained using the device-class-specific StandardScaler fitted only on benign training observations. Larger values therefore indicate a more extreme single-feature deviation from the corresponding benign device-class profile. It has no fitted detector state beyond the StandardScaler. This comparator is deliberately simple and uses the same benign validation calibration as the learned models.

For every detector and device class, the benign validation-score distribution is retained for calibration. Let *s* denote the raw anomaly score of a test observation and let $s_{1}^{\left(v\right)},\ldots ,s_{n_{v}}^{\left(v\right)}$ denote the corresponding benign validation scores. The empirical validation percentile is defined as

(7) $$p\left(s\right)=\frac{1}{n_{v}}\sum_{j=1}^{n_{v}}I\left(s_{j}^{\left(v\right)}\leq s\right),$$

where larger values indicate a greater deviation from the benign calibration distribution. The decision threshold is set at the 99th percentile. The percentile represents a relative rank with respect to benign validation behavior and should not be interpreted as a probability of compromise. This definition corresponds to the upper empirical cumulative rank: validation observations tied with the test score are included through the ≤ relation, so identical raw scores receive the same calibrated percentile. An observation is classified as anomalous when p(s)>0.99 in the baseline configuration.

Because the empirical percentile is bounded at 1.0, observations whose raw anomaly scores exceed the maximum benign validation score may receive the same percentile. For investigation-lead ordering, LITH therefore uses the calibrated percentile as the primary ranking key and the raw anomaly score as a secondary key for observations generated by the same detector and device class. Raw detector scores are not compared directly across device classes because the corresponding models and score distributions differ. When equal percentiles occur across different device classes, the observations are treated as equal-priority leads because their raw detector scores are not directly comparable; an operational implementation may use event time or a stable event identifier only to provide a deterministic display order.

Table 3 summarizes the detector configurations used in the controlled experiment.

### 3.6. Evaluation Protocol, Statistical Comparison, and Robustness Analysis

The controlled experiment is repeated as 10 Monte Carlo runs using independent random seeds 42–51. Within each device class, benign observations are split temporally into 60% training, 20% validation, and 20% test partitions. Thus, each run evaluates approximately 10,002 benign test observations together with all 5000 attack observations. No attack observation is used during model fitting, feature scaling, early stopping, or threshold calibration.

Detection performance is reported using precision, recall, F1-score, ROC-AUC, average precision (AP), and false-positive rate (FPR). ROC-AUC and AP are computed using the calibrated empirical percentile as the continuous ranking score, with attack observations treated as the positive class and larger scores indicating greater anomaly evidence. For each metric, the manuscript reports the mean and standard deviation across the 10 Monte Carlo runs; 95% confidence intervals are retained in the Supplementary Materials. Attack-specific, operating-point, feature-ablation, robustness, and hunt-lead analyses are reported primarily for Isolation Forest because it achieved the strongest overall threshold-free ROC-AUC in the baseline comparison. A single detector was retained for these secondary analyses to provide a consistent reference configuration and to avoid multiplying post hoc detector-by-analysis comparisons; this choice should not be interpreted as evidence that Isolation Forest is universally superior to the other evaluated detectors. The ablation compares network-only features (1–5), system-only features (6–8), and the combined eight-feature vector.

Because the baseline test set contains approximately 5000 attack observations and 10,002 benign observations, its attack prevalence is approximately 33.3%, which is substantially higher than may occur in operational monitoring. As precision depends on prevalence, we additionally report prevalence-adjusted positive predictive value (PPV) at lower illustrative attack prevalences. For attack prevalence π, expected PPV is calculated as

(8) $$\mathrm{PPV}\left(\pi \right)=\frac{\mathrm{TPR}\pi }{\mathrm{TPR}\pi +\mathrm{FPR}(1-\pi )}.$$

We report values for π=1% and π=0.1%. These prevalence levels are used as illustrative low-base-rate operating conditions rather than claims about a universal IoT attack prevalence.

Because the detector results are paired by seed, three planned paired *t*-tests compare One-Class SVM F1-score with Isolation Forest, the autoencoder, and Max-Z. Family-wise multiplicity is controlled using the Holm procedure, and Cohen’s $d_{z}$ is reported as a matched-sample effect size. These tests are treated as supportive comparisons rather than evidence of universal detector superiority.

Because only 10 matched Monte Carlo seeds are available, the distribution of paired F1-score differences is additionally assessed using the Shapiro–Wilk test. As a non-parametric robustness check, the same three detector comparisons are also evaluated using two-sided Wilcoxon signed-rank tests, with Holm correction applied across the three Wilcoxon comparisons.

A separate paired comparison is also performed between the network-only and combined Isolation Forest feature sets because the ablation conclusion is based on matched Monte Carlo seeds. The comparison reports the paired mean F1 difference, its 95% confidence interval, the paired *t*-test *p*-value, and Cohen’s $d_{z}$.

To test whether performance depends on a single hand-selected separation between benign and malicious feature distributions, a sensitivity experiment varies two factors while retaining benign-only fitting and validation calibration. Injected attack strength is set to 0.25, 0.50, 0.75, or 1.00 of the full attack deviation by linearly interpolating each attack vector between its benign-like base and full-strength target. Benign variability is multiplied by 1.00, 1.25, or 1.50; this multiplier increases both stochastic feature variation and the sinusoidal drift amplitude. For each variability level, an Isolation Forest bundle is refitted for every seed and then evaluated on matched attack-strength variants. This creates 12 conditions across the same 10 Monte Carlo seeds without tuning on attack labels.

A separate operating-point sensitivity analysis evaluates Isolation Forest at benign-validation percentile thresholds of 95, 97.5, 99, and 99.5. For each threshold, overall precision, recall, F1, FPR, exfiltration recall, and the number of benign investigation leads per 10,000 benign test observations are reported. Leads are normalized per 10,000 observations rather than per day because the feature-level benchmark does not impose a wall-clock aggregation-window duration.

To evaluate the analyst-facing ranking stage directly, we additionally compute precision@k and recall@k for Isolation Forest hunt-lead queues at budgets of 50, 100 and 500 leads per 10,000 observations. Because raw anomaly scores are used as secondary tie-breakers only within the same detector and device class, ranking is evaluated separately within each device-class-specific queue rather than by comparing raw scores across device classes. Within each queue, leads are ordered first by calibrated empirical percentile and then by raw anomaly score. For a queue containing *N* observations, a normalized budget *k* per 10,000 observations corresponds to round(kN/10,000) selected leads. True-positive and selected-lead counts are then pooled across the three device-class queues for each Monte Carlo seed, and precision@k and recall@k are summarized across the 10 seeds.

The contextual “changed indicators” attached to a hunt lead are not model-based feature attributions. They are defined as the four features with the largest absolute standardized deviations, $|z_{i}|$, from the corresponding device-class benign training profile. A fixed top-four rule is used to keep the analyst-facing record compact and is not selected using attack labels. For true-positive synthetic leads, generator-consistency fidelity is measured as the fraction of these four reported indicators that belong to the set of features intentionally modified by the corresponding attack generator. We also report the proportion of true-positive leads for which all four reported indicators are generator-consistent.

### 3.7. Analytics-Side Computational Footprint

A representative seed-42 detector bundle was benchmarked on a Linux x86_64 environment exposing five Intel Xeon Platinum 8573C logical CPU cores and approximately 5.81 GB of visible random-access memory (RAM). The software stack was Python 3.13.5, NumPy 2.3.5, pandas 2.2.3, SciPy 1.17.0, scikit-learn 1.8.0, and PyTorch 2.10.0+cpu. For each learned detector family, the three device-class-specific model files were serialized without compression; the Max-Z comparator has no detector state beyond the excluded scaler.

Latency measurements were performed using a single inference thread. Each timed repetition processed three vectorized batches of 1000 observations, 1 batch for each device class, for a total of 3000 observations per repetition. Then, 10 untimed warm-up repetitions were executed before 100 timed repetitions. The reported latency is the median wall-clock time normalized to 1000 observations. The timed path includes device-class-specific standardization and raw anomaly-score computation, but excludes the subsequent empirical-percentile lookup. These measurements characterize analytics-side inference only; they are not endpoint CPU, memory, or energy measurements.

### 3.8. Limited External Validation on CICIoT2023 Dataset

To examine whether the controlled-benchmark findings transfer beyond the author-designed generator, we conducted a limited external stress test using the Canadian Institute for Cybersecurity’s CICIoT2023 dataset [29]. CICIoT2023 was collected from an IoT environment containing 105 devices and includes 33 attacks grouped into 7 attack families. For computationally manageable evaluation, we used the public random_3way subsampled release of CICIoT2023, which preserves all 33 attack types and provides stratified training, calibration, and final-validation splits.

Because the processed CICIoT2023 representation does not provide direct counterparts for every LITH telemetry feature, the external experiment was restricted to three semantically aligned network features: Number (number of packets in the flow), AVG (average packet length), and Std (standard deviation of packet length). The CICIoT2023 protocolindicator was not treated as equivalent to LITH’s UDP-ratio feature, and no direct counterpart for unique-destination count was used. Therefore, this experiment should be interpreted as a limited network-feature stress test rather than a full external replication of the eight-feature LITH configuration.

The training split was restricted to benign observations for feature scaling and detector fitting. The test split was restricted to benign observations and used only for empirical-percentile calibration. The validation split, containing both benign and attack observations, was reserved for final evaluation. A StandardScaler was fitted only on benign training data. Isolation Forest used the same 100-tree configuration as the controlled benchmark and was fitted on 2000 benign observations for each of seeds 42–51. Max-Z was also evaluated using the same standardized-feature definition. Detection used the same strict 99th-percentile calibration rule, p(s)>0.99. Attack labels were not used for scaling, model fitting, or threshold calibration.

We report precision, recall, F1, ROC-AUC, average precision (AP), false-positive rate (FPR), and prevalence-adjusted PPV at illustrative attack prevalences of 1% and 0.1%. Because Max-Z has no stochastic model-fitting step under the fixed external split, its result is deterministic; repeated seeds affect only the Isolation Forest fitting sample and model.

## 4. Results

### 4.1. Overall Detection Performance and Simple Baseline

**Figure 3:**
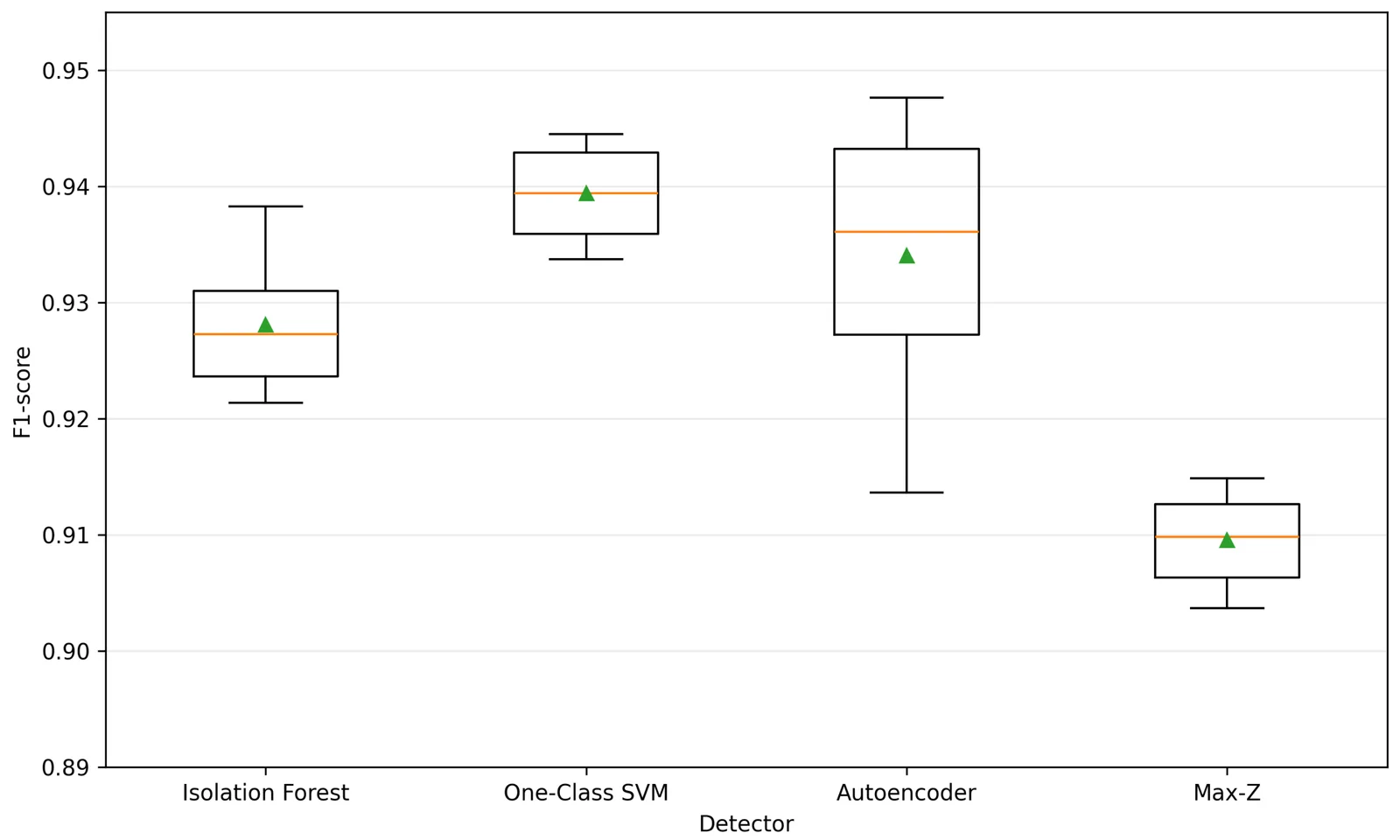
F1-score distributions for the three learned anomaly detectors and the Max-Z statistical baseline across 10 Monte Carlo runs. Boxes show the interquartile range, orange horizontal lines indicate medians, and green triangles indicate means. Whiskers indicate the range of non-outlying observations.

Table 4 reports the mean performance across the 10 Monte Carlo runs. At the common 99th-percentile calibration policy, One-Class SVM produced the highest mean thresholded F1-score among the evaluated detectors. This F1 comparison is specific to that operating point and should not be interpreted as an operating-point-independent ranking of the detectors. Using continuous decision-threshold-independent ranking metrics, Isolation Forest achieved the highest mean ROC-AUC and average precision (AP) in the baseline comparison. The Max-Z statistical baseline reached F1 = 0.909 ± 0.004 and ROC-AUC = 0.980 ± 0.001. Precision remained high across the four detectors, while the common 99th-percentile validation-calibrated decision rule produced benign test-set FPRs close to, but not exactly, 1%. This small deviation is expected because the threshold is calibrated on the benign validation-score distribution and then applied to a temporally later benign test segment that includes mild sinusoidal drift. Finite-sample empirical-percentile calibration and discrete score ties can also prevent the realized held-out FPR from equaling exactly 1%. Figure 3 shows the matched-run F1 distributions. All 30 autoencoder fits reached the predefined maximum of 25 epochs, indicating that the patience-based early-stopping condition was not activated before the epoch limit in these runs.

A training-size sensitivity analysis was also conducted to determine whether the fixed 2000-observation-per-class fitting budget materially affected the detector comparison. Using all available fitting observations changed Isolation Forest mean F1 from 0.9285 to 0.9251 and One-Class SVM from 0.9396 to 0.9390. The corresponding paired mean differences were −0.0034 (95% CI: −0.0090 to 0.0022) and −0.0006 (95% CI: −0.0029 to 0.0018), respectively. The autoencoder showed a larger reduction, from 0.9344 to 0.9210, with a paired mean difference of −0.0134 (95% CI: −0.0253 to −0.0016). Thus, the fixed training budget does not appear to artificially advantage Isolation Forest or One-Class SVM in this benchmark, although the autoencoder is more sensitive to fitting-set size under the unchanged architecture and training schedule. The expanded-data experiment should not be interpreted as evidence that smaller training sets are generally preferable.

A local hyperparameter sensitivity check was also performed without changing the baseline configurations. For Isolation Forest, mean F1 was 0.9204±0.0088, 0.9285±0.0040, and 0.9319±0.0046 for 50, 100, and 200 trees, respectively. Relative to the 100-tree baseline, the mean F1 differences were −0.0081 for 50 trees and +0.0034 for 200 trees. For the autoencoder, bottleneck widths of 2, 3, and 4 produced mean F1-scores of 0.9376±0.0070, 0.9344±0.0109, and 0.9406±0.0034, respectively. Thus, modest changes around the reported configurations did not qualitatively alter detector performance. These analyses are reported as post hoc sensitivity checks rather than as a basis for re-selecting hyperparameters from attack-labeled test results.

The precision values in Table 4 correspond to the experimental attack prevalence of approximately 33.3% and should not be interpreted as operational positive predictive values under lower attack base rates. Using each detector’s observed TPR and FPR, the expected PPV at an illustrative 1% attack prevalence is approximately 0.464 for Isolation Forest, 0.450 for One-Class SVM, 0.452 for the autoencoder, and 0.433 for Max-Z. At 0.1% prevalence, the corresponding values decrease to approximately 0.079, 0.075, 0.075, and 0.070, respectively. This base-rate effect is important for an analyst-facing system because even a low FPR can produce many benign investigation leads when true attacks are rare.

### 4.2. Matched-Seed Detector Comparisons

Table 5 summarizes the three paired comparisons across the matched Monte Carlo runs. One-Class SVM achieved a mean F1-score 0.0111 higher than Isolation Forest (95% CI: 0.0067 to 0.0155; Holm-adjusted $p=5.49\times 10^{-4}$; $d_{z}=1.82$). Its advantage over Max-Z was larger, with a mean difference of 0.0301 (95% CI: 0.0257 to 0.0346; Holm-adjusted $p=2.66\times 10^{-7}$; $d_{z}=4.88$). The difference between One-Class SVM and the autoencoder remained small (0.0051) and was not statistically significant after Holm correction (p=0.240; 95% CI: −0.0041 to 0.0144; $d_{z}=0.40$). Therefore, at the common 99th-percentile operating point, One-Class SVM had the highest mean F1-score among the evaluated detectors, although its difference from the autoencoder was not statistically significant. This thresholded comparison is operating-point dependent and is not used to claim an intrinsic detector ordering. The operating-point analysis demonstrates that F1 can change as the calibration threshold changes; consequently, ROC-AUC and AP are used as complementary continuous-score metrics when interpreting detector performance independently of a single threshold.

The Shapiro-Wilk tests applied to the paired F1-score differences did not provide strong evidence against normality for OCSVM versus Isolation Forest (p=0.223), OCSVM versus the autoencoder (p=0.061), or OCSVM versus Max-Z (p=0.281). Given the small sample of 10 matched seeds, we also repeated the comparisons using two-sided Wilcoxon signed-rank tests. After Holm correction, the Wilcoxon results supported the same qualitative conclusions: OCSVM differed from Isolation Forest (p=0.0059) and Max-Z (p=0.0059), while the difference from the autoencoder remained non-significant (p=0.4316).

### 4.3. Attack-Specific Isolation Forest Performance

Table 6 shows Isolation Forest performance for each attack type against the same benign test population. DDoS and reconnaissance were detected with very high recall and F1 because the injected behaviors create large deviations in packet rate, protocol mix, and/or destination count. Among the three evaluated attack–device configurations, the camera-based exfiltration configuration produced the lowest thresholded detection performance, with mean recall of 0.655 and F1 of 0.763 despite an ROC-AUC of 0.968. This comparison should not be interpreted as showing that exfiltration is intrinsically harder to detect than DDoS or reconnaissance, because attack type, device profile, and perturbation magnitude vary together in the present benchmark. The result shows that a strong ranking metric does not guarantee equally strong binary detection at a strict 99th-percentile threshold when the evaluated attack configuration overlaps more strongly with its corresponding benign profile.

### 4.4. Operating-Point Sensitivity

**Figure 4:**
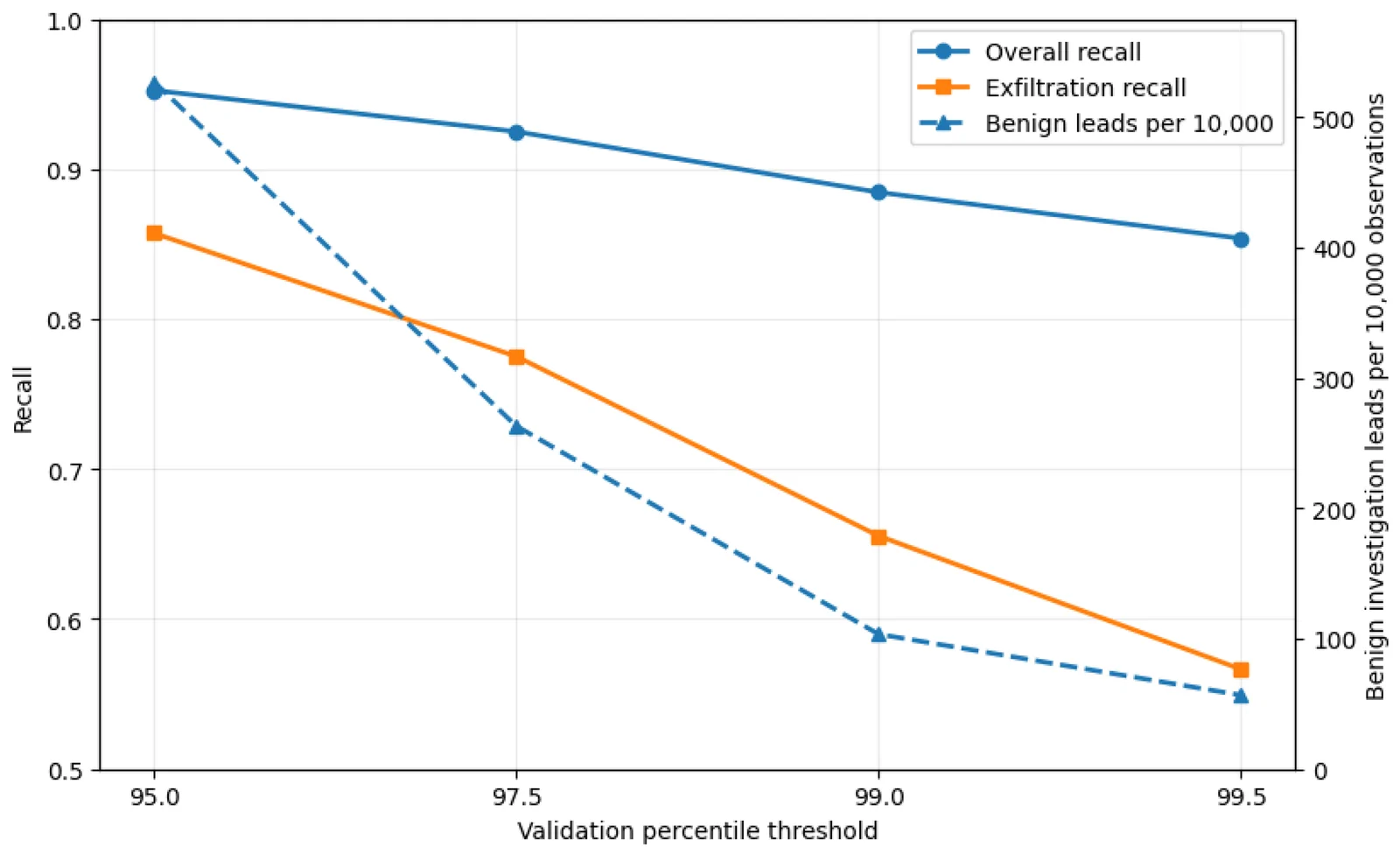
Isolation Forest operating-point sensitivity across four validation-percentile thresholds. The thresholds are shown as equally spaced discrete operating points to avoid visually exaggerating slope differences caused by unequal numeric intervals. Overall and exfiltration recall are shown on the left axis, while normalized benign investigation-lead volume is shown on the right axis to illustrate the detection–workload trade-off. Blue circles connected by a solid line represent overall recall, orange squares connected by a solid line represent exfiltration recall, and blue triangles connected by a dashed line represent benign investigation leads per 10,000 observations. Connecting lines serve as visual guides between the evaluated operating points.

The 99th-percentile threshold used in the baseline results is a policy operating point rather than an intrinsic optimum. Table 7 and Figure 4 quantify the Isolation Forest trade-off across four benign-validation percentile thresholds. Lowering the threshold from 99 to 97.5 increased overall recall from 0.884 to 0.925 and exfiltration recall from 0.655 to 0.775, while increasing FPR from 1.03% to 2.63%. Expressed as normalized analyst-facing volume, this corresponds to approximately 103 versus 263 benign investigation leads per 10,000 benign observations.

A 95th-percentile threshold increased exfiltration recall further to 0.857 but generated approximately 527 benign leads per 10,000 observations. Conversely, the 99.5th percentile reduced benign leads to approximately 57 per 10,000 observations but lowered exfiltration recall to 0.567.

The prevalence-adjusted analysis further illustrates the base-rate effect. At the baseline 99th-percentile operating point, expected PPV is approximately 0.464 at an illustrative 1% attack prevalence and 0.079 at 0.1% prevalence. Increasing the threshold to the 99.5th percentile raises the corresponding expected PPV values to approximately 0.603 and 0.131 because of the lower false-positive rate, but this comes at the cost of reduced attack recall. Thus, threshold selection in deployment must account not only for detector sensitivity and benign lead volume, but also for the expected attack base rate. These results show that threshold selection should be treated as an operational policy choice that balances missed anomalies against investigation workload; the controlled benchmark does not define a universal SOC-optimal threshold.

### 4.5. Feature Ablation

**Figure 5:**
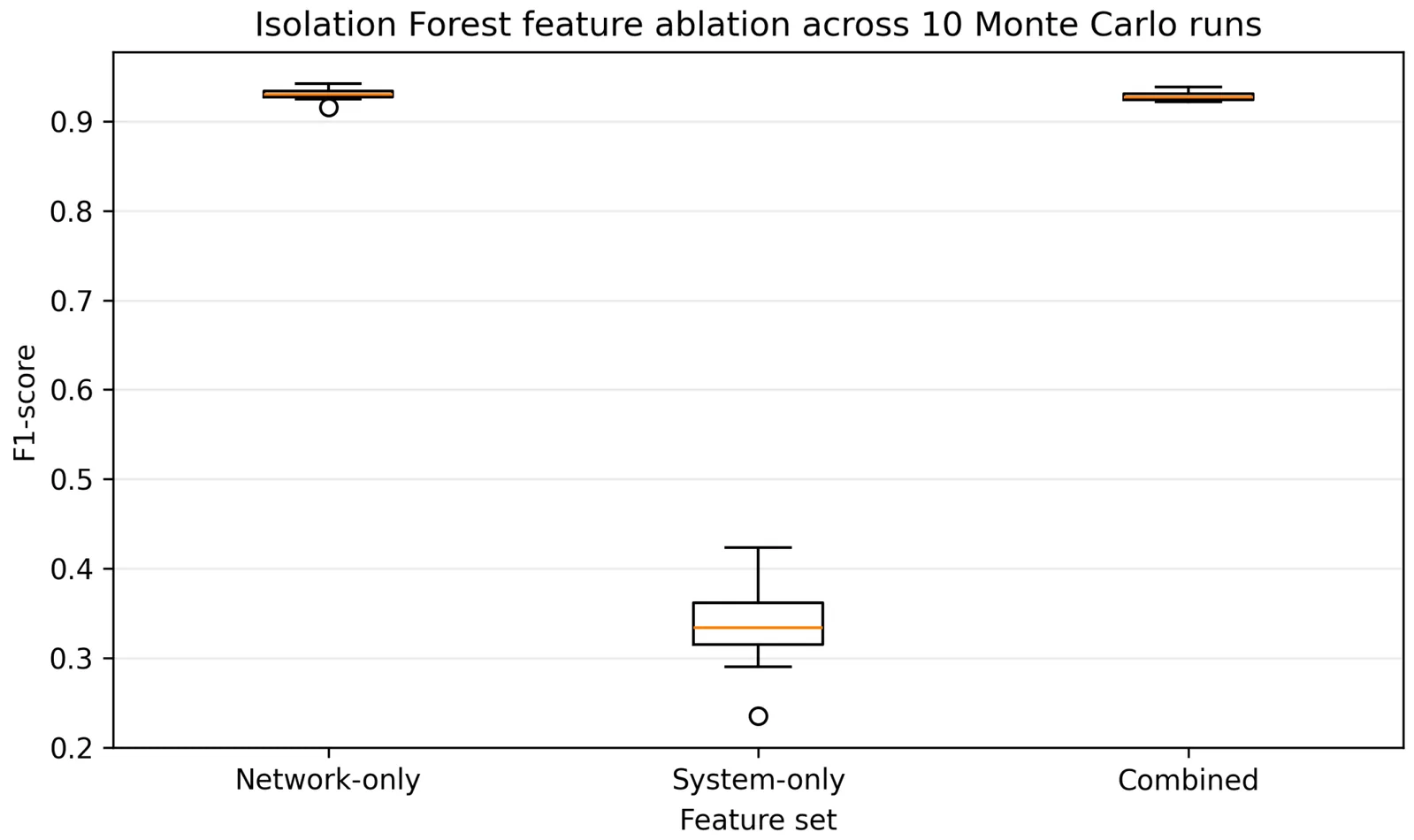
Isolation Forest F1-score distributions for network-only, system-only, and combined feature sets across 10 Monte Carlo runs. Boxes show the interquartile range, orange horizontal lines indicate medians, whiskers indicate the range of non-outlying observations, and open circles denote outlying runs.

The Isolation Forest ablation results are shown in Table 8 and Figure 5. Network-only telemetry produced F1= 0.929±0.009, which was numerically very close to the combined feature set (0.928±0.004), while system-only telemetry performed substantially worse (F1 = 0.334±0.050).

This ablation result is conditioned by the benchmark design: all three evaluated attack configurations directly perturb multiple network-derived features, while the available system indicators provide weaker or less distinctive evidence for these particular scenarios. The near-parity of the network-only and combined configurations should therefore not be generalized to attack classes whose principal evidence is host-local or process-level.

The matched-seed comparison confirms that the difference between the network-only and combined configurations is negligible. Network-only exceeded the combined configuration by a mean F1 difference of only 0.0007 (95% CI: −0.0072 to 0.0087), which was not statistically significant (paired *t*-test, p=0.843; Cohen’s $d_{z}=0.065$). The experiment therefore does not establish that adding the system features degrades Isolation Forest performance. Instead, it indicates that the five network-derived features are sufficient to retain essentially the same detection performance for the three evaluated synthetic attack configurations. This finding supports gateway- or tap-based monitoring for the evaluated scenarios, while host-level telemetry remains potentially useful for other attack types and contextual investigation.

### 4.6. Sensitivity to Attack Strength and Benign Variability

**Figure 6:**
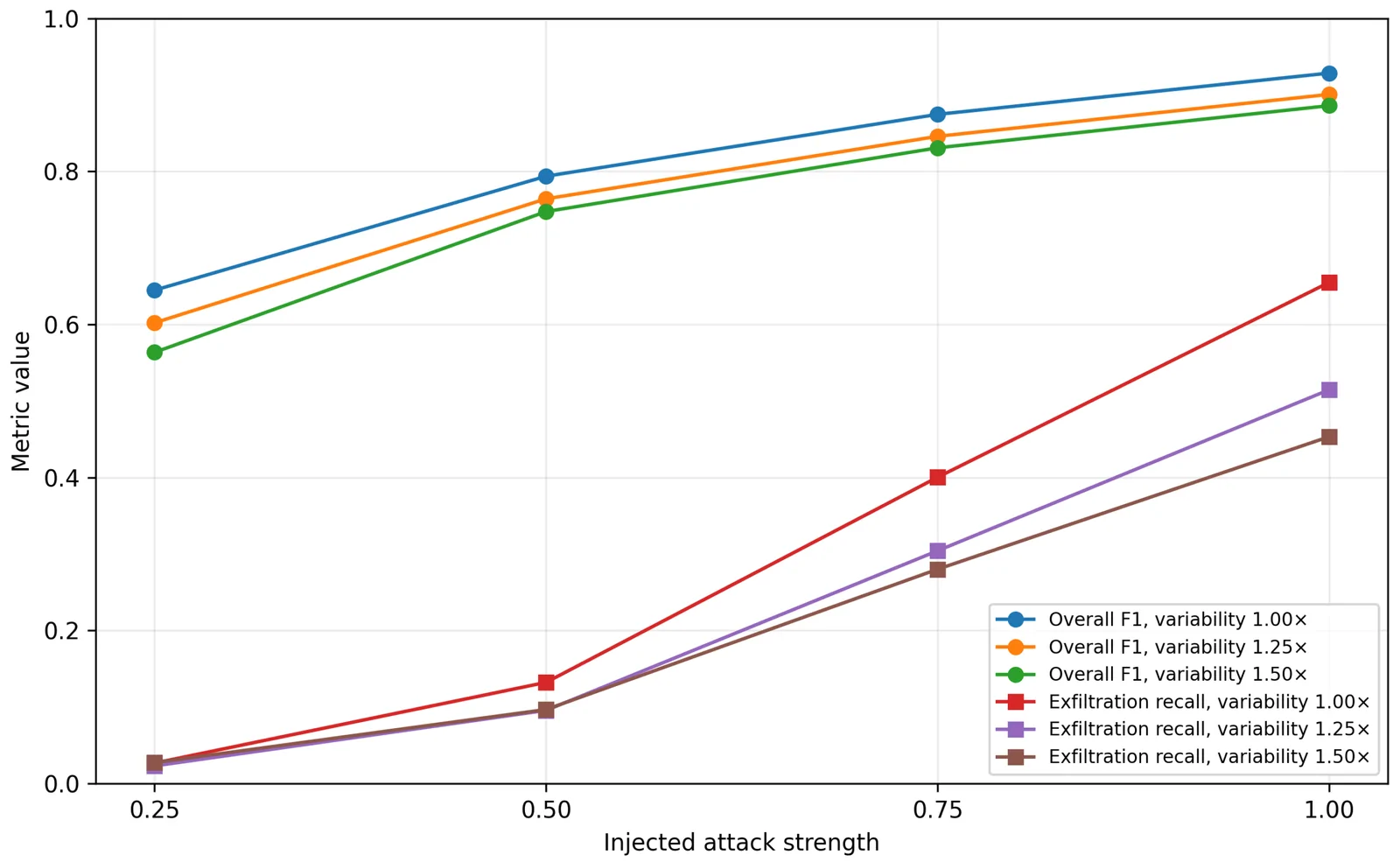
Isolation Forest sensitivity to injected attack strength and benign variability. Overall F1 is shown together with exfiltration recall to expose attack-specific behavior that can be obscured by aggregate performance. In particular, exfiltration recall falls to approximately 0.02–0.03 at quarter-strength attacks across the evaluated benign-variability levels.

Figure 6 and Table 9 show that Isolation Forest performance degrades progressively as the injected attack becomes less distinct and the benign distribution becomes more variable. Under baseline variability, mean overall F1 decreased from approximately 0.928 at full attack strength to 0.645 at quarter strength. With benign variability increased to 1.50 times the baseline level, the corresponding values were approximately 0.886 and 0.564.

The attack-specific breakdown reveals that the aggregate F1 values mask a substantially greater loss of sensitivity for weak exfiltration behavior. At attack strength 0.25 and baseline variability, mean recall was 0.870 for DDoS and 0.562 for reconnaissance, but only 0.027 for the evaluated camera-based exfiltration configuration. Exfiltration recall remained similarly low at increased benign variability, reaching 0.023 at 1.25 times baseline variability and 0.027 at 1.50 times baseline variability. Thus, the quarter-strength aggregate result should not be interpreted as indicating comparable sensitivity across attack configurations.

The progressive decline across the sensitivity grid is consistent with detector performance responding to separation strength rather than succeeding only at one chosen attack magnitude. At the same time, the very low recall for weak exfiltration demonstrates an important limitation of the present detector and threshold configuration.

### 4.7. Analytics-Side Computational Footprint

Table 10 reports the serialized detector state (excluding scaler and validation-score arrays) and median inference time per 1000 observations on the evaluation host. The shallow autoencoder had the smallest learned bundle and fastest learned-model inference, followed by One-Class SVM. Isolation Forest remained modest for a central analytics service but required a larger bundle because it stores 100 trees per device profile. Max-Z requires no fitted detector state beyond the excluded StandardScaler and was fastest in this benchmark. These values should not be interpreted as endpoint resource measurements.

### 4.8. Case Study: Converting a DDoS Anomaly into a Hunt Lead

An illustrative high-score seed-42 DDoS observation was selected to demonstrate how LITH separates anomaly detection from threat confirmation. The selected observation has the largest raw Isolation Forest anomaly score among the seed-42 DDoS samples and reaches the 100th empirical validation percentile, above the 99th-percentile decision threshold.

Percentile saturation occurs for multiple high-scoring observations in this run: 1658 of 1667 DDoS observations, 322 of 1667 exfiltration observations, and 1291 of 1666 reconnaissance observations map to 1.000, compared with 4 of 10,002 benign test observations. This saturation motivates retaining the raw anomaly score as the secondary ordering key within the same detector and device class.

For the selected DDoS observation, the four largest absolute standardized deviations from the sensor’s benign profile are UDP ratio, unique destination count, packet-size standard deviation, and mean packet size. Table 11 shows the corresponding analyst-facing record. The framework does not label the event as Mirai or another specific malware family; it creates a high-priority investigation lead because the anomaly score and concurrent behavioral deviations justify further analysis.

To complement the extreme high-score DDoS example, two additional seed-42 observations were selected using boundary-based rules rather than manual inspection. The borderline exfiltration example is the detected exfiltration observation with the smallest calibrated percentile above the baseline p(s)>0.99 decision threshold. The benign false-positive example is the benign test observation whose calibrated percentile lies closest above the same threshold. These cases illustrate the types of ambiguous leads that an analyst would encounter near the operational decision boundary. Table 12 presents the two boundary-case investigation leads and their associated behavioral indicators.

The borderline exfiltration observation exceeds the decision boundary only slightly, despite showing several deviations from the camera’s benign profile. Therefore, it represents a true-positive lead for which additional contextual correlation would still be necessary before any security action. Conversely, the benign sensor observation also crosses the threshold because of unusual but non-malicious behavior, most notably its elevated destination count and packet-size variability. This false-positive example demonstrates why LITH treats threshold crossings as investigation leads rather than confirmed compromises and why automated containment is intentionally excluded from the current framework.

### 4.9. Hunt-Lead Ranking and Indicator Fidelity

To evaluate the anomaly-to-hunt-lead stage directly, Table 13 reports precision@k and recall@k for Isolation Forest at normalized queue budgets of 50, 100, and 500 leads per 10,000 observations. Ranking was performed separately within each device-class-specific queue using the calibrated empirical percentile as the primary key and the raw anomaly score as the secondary key. Counts were then pooled across the three device-class queues within each Monte Carlo seed.

At the smallest two queue budgets, all selected leads were attack observations in every run, yielding mean precision@50 and precision@100 of 1.000. At the larger budget of 500 leads per 10,000 observations, mean precision remained 0.9993 ± 0.0011. Recall increased with queue depth, from 0.0150 at 50 leads per 10,000 observations to 0.1499 at 500 leads per 10,000 observations. These results indicate that, within the controlled benchmark, the highest-ranked Isolation Forest leads are strongly concentrated among the injected attack observations. They should not be interpreted as expected operational queue precision because the benchmark is synthetic and has a substantially higher attack prevalence than typical low-base-rate monitoring environments.

The changed-indicator analysis provides a complementary check of the context attached to the ranked leads. Across 44,222 true-positive leads generated at the baseline 99th-percentile operating point, the mean generator-consistency fidelity of the four reported indicators was 0.9948. All four reported indicators belonged to the set of features intentionally modified by the corresponding synthetic attack generator in 97.91% of true-positive leads.

The result varied slightly by attack configuration. Mean indicator fidelity was 0.9995 for DDoS, 1.0000 for exfiltration, and 0.9866 for reconnaissance. The corresponding proportions for which all four reported indicators were generator-consistent were 99.81%, 100.00%, and 94.62%, respectively. The perfect result for the exfiltration configuration should be interpreted cautiously because that generator modifies all eight evaluated features. More generally, this fidelity measure assesses consistency with known synthetic perturbations rather than causal explanation accuracy or model-based feature attribution. Table 14 summarizes generator-consistency fidelity for the evaluated attack configurations.

### 4.10. External Network-Feature Stress Test on CICIoT2023

Table 15 summarizes the limited external evaluation. The results differ substantially from those obtained under the controlled synthetic benchmark. At the 99th-percentile operating point, Isolation Forest achieved a mean ROC-AUC of 0.625±0.027 but extremely low recall (0.00036±0.00009), yielding an F1-score of only 0.00072±0.00018. In contrast, the deterministic Max-Z baseline achieved a recall of 0.702, F1 of 0.824, ROC-AUC of 0.835, and AP of 0.971.

These results therefore do not reproduce the detector ranking observed in the controlled synthetic benchmark. In particular, the external experiment shows that the learned Isolation Forest operating point does not transfer reliably under the reduced three-feature representation and the substantially different benign and attack distributions of CICIoT2023.

Max-Z performance was also strongly attack-family dependent. Recall was approximately 1.000 for DDoS, DoS, and Mirai traffic, while recall was 0.0020 for reconnaissance, 0.0003 for spoofing, 0.0035 for web-based attacks, and 0.0054 for brute-force attacks. Thus, the overall Max-Z performance is driven primarily by attack families that generate large deviations in the three retained traffic-volume and packet-size features.

The external experiment should consequently be interpreted as a stress test of limited feature transfer rather than evidence of full cross-dataset generalization. Nevertheless, it provides independent evidence that strong behavioral deviations can be surfaced using compact telemetry while also showing that both detector choice and telemetry coverage materially affect performance outside the controlled generator.

## 5. Discussion

### 5.1. Interpretation of the Experimental Findings

The evaluation design excludes attack observations from model fitting and threshold calibration, uses physically discrete count-valued features, reports performance across 10 Monte Carlo runs, and includes both a simple statistical comparator and an explicit robustness grid. Under the baseline protocol, all learned model families maintain high precision with mean FPR close to the approximately 1% validation-calibration target. One-Class SVM is numerically strongest by thresholded F1, while Isolation Forest provides the strongest ROC-AUC. The matched-seed tests show that the One-Class SVM advantage over Isolation Forest is statistically supported under this benchmark, but its difference from the autoencoder is not. The strong Max-Z baseline deserves particular attention. In the corrected baseline experiment, Max-Z achieved a mean F1-score of approximately 0.909, compared with 0.928 for Isolation Forest, while its mean ROC-AUC was 0.980 compared with 0.989 for Isolation Forest. Thus, a single extreme standardized feature captures a substantial fraction of the separability present in this controlled benchmark. This is consistent with the benchmark design, in which several injected attacks create relatively large shifts in individual network features.

The learned detectors nevertheless provide additional value by modeling multivariate structure rather than relying solely on the single most extreme feature. Their higher F1, ROC-AUC, and AP values indicate that combining information across features improves both thresholded detection and continuous ranking under the evaluated conditions. However, the relatively modest gap between Max-Z and the learned models also limits the strength of conclusions that can be drawn from the synthetic benchmark alone. It suggests that simple statistical baselines may be competitive when attacks produce strong univariate deviations, while the benefit of learned multivariate models should be evaluated more critically on less structured and real-world data.

The results should nevertheless be interpreted in the context of a controlled telemetry generator. They demonstrate that the LITH workflow is reproducible and that device-class-specific benign baselines can distinguish the injected behaviors under documented distributional assumptions. They do not establish performance on physical devices, raw packet traces, encrypted production networks, or previously unseen real-world malware.

The external CICIoT2023 stress test further qualifies the conclusions drawn from the controlled benchmark. In the external three-feature setting, the synthetic detector ranking did not persist: Max-Z substantially outperformed Isolation Forest, while its detection capability was concentrated primarily in DDoS, DoS, and Mirai traffic. This result supports the concern that the stronger performance observed for learned detectors in the controlled generator should not be interpreted as evidence of detector superiority across unseen IoT environments. It also suggests that simple standardized deviations can transfer effectively when attacks produce large univariate shifts, while more subtle attack families require richer behavioral context.

### 5.2. Interpretation of the Evaluated Exfiltration Configuration

The attack-specific analysis reveals a useful limitation rather than an intrinsic ordering of attack difficulty. In the present benchmark, DDoS is evaluated against the environmental-sensor profile, reconnaissance against the actuator profile, and exfiltration against the smart-camera profile. The camera-based exfiltration configuration produces the lowest recall because its injected behavior is intentionally closer to the corresponding benign camera distribution, while the evaluated DDoS and reconnaissance configurations produce larger multifeature deviations from their respective benign profiles.

Accordingly, the lower exfiltration performance cannot be attributed to attack type alone. Device-profile variability and perturbation magnitude are confounded with attack type in the current design. A controlled comparison of intrinsic attack-type difficulty would require applying each attack type across multiple device profiles and at matched perturbation levels. Isolation Forest still ranks exfiltration observations well (ROC-AUC 0.968), but the strict 99th-percentile threshold yields recall of only 0.655. This should not be interpreted as adequate production exfiltration protection. Instead, it exposes the operational trade-off between a low benign alert rate and sensitivity to subtle behavior. The operating-point analysis shows that moving to the 97.5th percentile raises exfiltration recall to 0.775 while more than doubling the normalized benign lead volume; further lowering the threshold improves recall again but produces substantially more analyst-facing leads. Multistage detection, contextual enrichment, or deployment-specific threshold policy would therefore be needed for stronger exfiltration coverage.

### 5.3. Implications of the Feature Ablation

The ablation study identifies two practical deployment modes. A network-observer mode uses only gateway- or tap-visible features, while an augmented mode adds optional host telemetry when it is available. For the three simulated attacks, network-only telemetry performs essentially as well as the combined feature set, while system-only telemetry is inadequate. This finding supports the practical use of network taps, smart switches, or gateways when constrained devices cannot expose host metrics. It also suggests that CPU, memory, and process count should be treated as optional context rather than mandatory detection inputs for these attack types. The result may not generalize to attacks whose primary footprint is local computation, persistence, or memory abuse; such scenarios should be included in future physical-device testing.

### 5.4. Robustness Beyond a Single Synthetic Separation

A controlled feature-level benchmark necessarily encodes assumptions about how attacks perturb benign behavior. The sensitivity study was added specifically to test whether the reported detection performance depends on one favorable separation level. F1 decreases consistently as attack strength is reduced and benign variability is increased, from 0.928 under the baseline full-strength condition to 0.564 under quarter-strength attacks with 1.50× benign variability. This behavior is desirable in the sense that it exposes a measurable difficulty gradient rather than producing uniformly near-perfect scores. However, the experiment remains synthetic: changing attack strength by interpolation is not equivalent to observing naturally occurring low-and-slow attacks, and increasing generator variance cannot reproduce every source of benign heterogeneity. The robustness results reduce, but do not eliminate, the risk of circularity inherent in feature-level simulation.

### 5.5. From Anomaly Detection to Hunt-Lead Generation

LITH’s central conceptual contribution is the separation of detection from investigation. Automated anomaly detection answers the question “Which observations differ from this device class’s benign profile?” A full threat-hunting process additionally involves hypothesis generation, active exploration, contextual correlation, and iterative analyst refinement. LITH does not implement that complete lifecycle. Instead, it operationalizes the preceding anomaly-to-hunt-lead stage by calibrating anomaly scores to device-class-specific validation percentiles and using that percentile as the primary ranking signal, with the raw anomaly score retained as a secondary tie-breaker within the same detector and device class. The illustrative high-score DDoS lead demonstrates how score, device context, and changed features can be packaged to initiate or guide analyst- or SIEM-supported hunting without claiming autonomous malware attribution. The current LITH implementation operates on device-class-level behavioral telemetry and is therefore most suited to anomalies that cause an observable deviation in the monitored behavior of one or more individual devices, such as changes in traffic volume, protocol mix, destination diversity, packet-size behavior, or optional host-resource indicators. Its scope does not extend to semantic conflicts among automation rules or coordinated interactions whose individual device behaviors remain locally normal. Such anomalies may arise from dependencies among devices, data sources, actuators, and trigger–action rules and generally require cross-component, causal, or rule-level reasoning beyond the present telemetry model. Prior work has highlighted anomaly detection across multiple IoT layers [24] and has specifically addressed interference and conflict detection among automation rules and IIoT control policies [25,26]. LITH should therefore be viewed as complementary to, rather than a replacement for, cross-device and rule-interaction analysis. In a broader deployment, these forms of reasoning could be implemented at the SIEM or correlation layer above the device-class-specific LITH scores.

### 5.6. Scalability and Deployment Considerations

The hybrid architecture keeps the main detector away from constrained endpoints and supports a network-observer mode for devices that cannot host agents, with host telemetry treated as optional augmentation rather than a requirement for the evaluated attack types. The analytics-side benchmark indicates that all detector bundles are small enough for centralized or edge-hosted analytics on the evaluated general-purpose CPU, and Max-Z demonstrates the lower bound of a nearly stateless comparator. These results support analytics-side feasibility but are not substitutes for endpoint measurements. At larger scale, device populations can be partitioned across analytics nodes, edge aggregation can reduce upstream telemetry, and device-type profiles can be maintained separately to accommodate heterogeneous normal behavior.

In this study, the term “lightweight” refers to the architectural use of compact telemetry, optional host instrumentation, and offloaded analytics rather than to an experimentally demonstrated endpoint CPU, memory, or energy footprint.

### 5.7. Limitations and Future Validation

The primary evaluation in this study remains a controlled synthetic benchmark at the behavioral-feature level. Although the revised study now includes a limited external network-feature stress test using CICIoT2023, that experiment uses only three directly aligned network features and does not reproduce LITH’s full eight-feature, device-class-specific configuration. It therefore provides partial evidence about cross-dataset transfer rather than full external validation of the complete framework. The controlled generator nevertheless remains useful for reproducibility, benign-only fitting, leakage-free calibration, matched-seed statistical testing, comparison with the Max-Z baseline, and systematic attack-strength and benign-variability sensitivity analysis. In particular, the attack perturbations are generated within the same feature space used by the anomaly detectors, and the strong performance of the simple Max-Z baseline confirms that part of the separability in the benchmark arises from these controlled feature shifts. Consequently, the reported detection values should be interpreted as proof-of-concept evidence for the LITH anomaly-to-hunt-lead workflow rather than estimates of expected operational performance.

LITH may also be vulnerable to behavioral mimicry, where an adversary deliberately keeps malicious activity within the learned benign profile to reduce anomaly scores. In addition, legitimate changes such as firmware updates, new services, configuration changes, or evolving usage patterns may shift normal behavior and increase false-positive rates unless baselines are periodically reviewed and updated.

The experimental test set also uses an intentionally high attack prevalence of approximately 33.3%; therefore, the directly observed precision values should not be interpreted as expected operational precision in low-base-rate environments, as demonstrated by the prevalence-adjusted PPV analysis.

Second, the feature generator does not model a physical aggregation-window duration, packet order, encrypted-flow timing, energy consumption, or endpoint collection overhead. Consequently, the operating-point analysis reports normalized benign leads per 10,000 observations rather than leads per day; translating these values to SOC workload requires a real observation cadence and deployment-specific analyst capacity. Third, the attack set remains limited to three scenarios and is dominated by network-visible effects, so the value of host-level features remains under-tested. Fourth, the analytics benchmark was performed on a general-purpose CPU rather than an embedded gateway. Fifth, the Monte Carlo replicates are repeated draws from the same stochastic generator, not independent real-world deployments. Finally, the current system implements anomaly-to-hunt-lead generation rather than the full threat-hunting lifecycle, and it ranks leads by anomaly percentile without learning a separate risk score from analyst feedback.

The limited CICIoT2023 external stress test provides an initial assessment of cross-dataset transfer but should not be interpreted as full external validation of LITH. Only three network features—packet count, mean packet length, and packet-length standard deviation—had sufficiently direct semantic correspondence with the processed public dataset. The experiment therefore does not reproduce the complete device-class-specific eight-feature telemetry configuration evaluated in the controlled benchmark.

Importantly, the external results also showed that the detector ranking observed in the controlled benchmark did not transfer uniformly. Under the three-feature CICIoT2023 setting, Max-Z substantially outperformed Isolation Forest, while its recall was concentrated primarily in DDoS, DoS, and Mirai traffic and remained low for several other attack families. This finding reinforces the need for broader external evaluation using independently collected IoT datasets with richer feature correspondence and device-level identity information.

Future work should therefore extend external validation to additional public and physical-device datasets, evaluate richer mappings of LITH telemetry, and test device-class-specific calibration under independently collected traffic. A physical heterogeneous testbed should additionally quantify endpoint and edge processing, upstream bandwidth, energy consumption, and real aggregation-window latency.

## 6. Conclusions

This paper presented LITH, a lightweight behavioral anomaly-to-hunt-lead framework that connects IoT telemetry, edge preprocessing, device-class-specific anomaly detection, and analyst-facing investigation support. The controlled benchmark uses discrete count-valued features, benign-only temporal training and validation, 10 Monte Carlo seeds, a simple statistical comparator, matched-seed significance testing, feature ablation, attack-strength/benign-variability sensitivity analysis, and operating-point analysis. At the common 99th-percentile operating point, One-Class SVM achieved the highest mean thresholded F1-score, although its difference from the autoencoder was not statistically significant after Holm correction. Because thresholded F1 depends on the selected operating point, this result should not be interpreted as an operating-point-independent detector ranking. Isolation Forest achieved the highest mean ROC-AUC and AP among the evaluated detectors. Isolation Forest detected the evaluated DDoS and reconnaissance configurations strongly but was less sensitive to the evaluated camera-based exfiltration configuration (F1 = 0.763 ± 0.016). Network-only telemetry retained essentially all combined-feature performance, while system-only telemetry was insufficient for the evaluated attacks.

A limited external stress test on CICIoT2023 using three semantically aligned network features further showed that the detector ranking observed in the controlled benchmark does not transfer uniformly. Max-Z achieved an F1-score of 0.824 and ROC-AUC of 0.835, while Isolation Forest did not transfer reliably at the fixed 99th-percentile operating point. These results reinforce the need to evaluate both detector choice and telemetry coverage across independently collected IoT environments.

The robustness grid showed that detection performance declines as injected attacks become weaker and benign behavior becomes more variable, rather than remaining uniformly high across all synthetic conditions. This finding strengthens the proof of concept while also clarifying its limits. LITH’s main contribution is an operational architecture that converts calibrated behavioral deviations into ranked investigation leads that can support threat hunting while keeping automated detection separate from confirmation. The threshold analysis further shows that deployment policy must explicitly balance subtle-attack recall against benign lead volume. The current results establish reproducible feasibility under controlled assumptions, not deployment readiness. Physical-device, packet-level, energy, bandwidth, aggregation-window, and real-world workflow studies remain necessary before LITH can be treated as a production IoT security system.

## Figures and Tables

**Table 1:** Positioning of LITH relative to representative recent IoT intrusion-detection and threat-hunting work. The comparison is qualitative because the studies use different datasets, deployment settings, and resource measurements. LITH’s deployment/resource characterization reflects architectural design and analytics-side measurements rather than validated endpoint or embedded-edge measurements.

Study	Deployment/Resource Focus	Detection Approach	Threat-Hunting Integration
Zachos et al. [14]	Strong; resource-constrained IoMT	Novelty/outlier detection with remote engine	Detection-focused
Almalawi [15]	Strong; lightweight IoT IDS	Data condensation and stable-feature selection	Detection-focused
Mutambik [12]	Moderate; flow-based IoT monitoring	Benign-behavior anomaly detection	Detection-focused
Yazdinejad et al. [19]	Deep-learning-oriented IIoT	LSTM and autoencoder ensemble	Explicit cyber-threat hunting
Jahromi et al. [20]	Privacy/scalability-oriented IIoT	Federated deep representation and ensemble	Explicit cyber-threat hunting
LITH (this work)	Qualified; hybrid endpoint-edge-central design with analytics-side footprint measured, but endpoint/edge resource cost not yet validated	Minimal telemetry and lightweight unsupervised scoring	Explicit anomaly-to-investigation workflow

**Table 2:** Behavioral feature set used in the controlled experiment.

No.	Feature	Source	Definition and Behavioral Rationale
1	Packet count	Network	Integer number of packets in the behavioral observation; captures traffic-rate changes.
2	Mean packet size	Network	Mean packet size in bytes; captures shifts in transfer behavior.
3	Packet-size standard deviation	Network	Standard deviation of packet size in bytes; distinguishes stable periodic traffic from burstier behavior.
4	Unique destination IP count	Network	Integer number of distinct destinations; highlights new peers or scanning.
5	UDP ratio	Network	UDP packets divided by total packets; captures protocol-mix shifts.
6	Central processing unit (CPU) utilization	System	Host CPU utilization percentage, when available.
7	Memory utilization	System	Host memory utilization percentage, when available.
8	Process count	System	Integer number of active processes, when available.

**Table 3:** Detector configurations used in the controlled experiment.

Detector	Configuration
Isolation Forest	100 estimators; max samples 256; all input features; contamination = auto; device-class-specific model; external 99th-percentile validation calibration.
One-Class SVM	RBF kernel; ν=0.1; γ = scale; tolerance = 0.001; 1000 MB cache; device-class-specific model.
Autoencoder	8-6-4-3-4-6-8; ReLU hidden layers; linear output; Adam (0.001); mean squared error (MSE) reconstruction loss; maximum 25 epochs; batch size 256; early-stopping patience 4.
Max-Z baseline	Maximum absolute device-standardized feature value; no fitted detector state beyond the StandardScaler.

**Table 4:** Overall performance across 10 Monte Carlo runs (mean ± standard deviation). Precision, recall, F1, and FPR are reported at the common 99th-percentile validation-calibrated operating point; ROC-AUC and AP are computed from the continuous calibrated ranking scores and do not depend on a single decision threshold.

Detector	Precision	Recall	F1	ROC-AUC	AP	FPR
Isolation Forest	0.977±0.002	0.884±0.008	0.928±0.004	0.989±0.001	0.983±0.002	0.0103±0.0012
One-Class SVM	0.976±0.002	0.906±0.008	0.940±0.004	0.987±0.001	0.982±0.002	0.0112±0.0009
Autoencoder	0.976±0.002	0.896±0.020	0.934±0.011	0.986±0.004	0.981±0.004	0.0110±0.0010
Max-Z baseline	0.974±0.004	0.853±0.008	0.909±0.004	0.980±0.001	0.966±0.002	0.0113±0.0017

**Table 5:** Planned paired F1-score comparisons across matched Monte Carlo seeds. Holm-adjusted *p*-values control the family-wise error rate across the three tests.

Comparison	Mean F1 Difference	95% CI	Holm-adjusted *p*	Cohen’s $d_{z}$
OCSVM vs. Isolation Forest	0.0111	0.0067 to 0.0155	$5.49\times 10^{-4}$	1.82
OCSVM vs. Autoencoder	0.0051	−0.0041 to 0.0144	0.240	0.40
OCSVM vs. Max-Z	0.0301	0.0257 to 0.0346	$2.66\times 10^{-7}$	4.88

**Table 6:** Isolation Forest attack-specific performance across 10 Monte Carlo runs (mean ± standard deviation).

Attack	Precision	Recall	F1	ROC-AUC	AP
DDoS botnet	0.942±0.006	1.000±0.000	0.970±0.003	1.000±0.000	0.998±0.002
Data exfiltration	0.914±0.008	0.655±0.024	0.763±0.016	0.968±0.003	0.882±0.011
Reconnaissance	0.941±0.006	0.998±0.002	0.969±0.003	1.000±0.000	0.996±0.002

**Table 7:** Isolation Forest operating-point sensitivity across 10 Monte Carlo runs (means). Benign leads are normalized per 10,000 benign test observations because the benchmark does not define a wall-clock observation rate. Expected PPV values are prevalence-adjusted estimates calculated from the observed overall recall (TPR) and FPR at illustrative attack prevalences of 1% and 0.1%.

Validation Percentile	Precision	Overall Recall	Overall F1	FPR	Exfiltration Recall	Benign Leads per 10,000	Expected PPV at 1%	Expected PPV at 0.1%
95.0	0.900	0.952	0.926	0.0527	0.857	527	0.154	0.018
97.5	0.946	0.925	0.935	0.0263	0.775	263	0.262	0.034
99.0	0.977	0.884	0.928	0.0103	0.655	103	0.464	0.079
99.5	0.987	0.854	0.915	0.00567	0.567	57	0.603	0.131

**Table 8:** Isolation Forest feature-ablation results across 10 Monte Carlo runs (mean ± standard deviation).

Feature Set	Precision	Recall	F1	ROC-AUC
Network-only	0.977±0.003	0.886±0.014	0.929±0.009	0.989±0.001
System-only	0.896±0.031	0.206±0.037	0.334±0.050	0.810±0.007
Combined	0.977±0.002	0.884±0.008	0.928±0.004	0.989±0.001

**Table 9:** Isolation Forest sensitivity to attack strength and benign variability across 10 Monte Carlo runs. The final three columns represent benign-variability multipliers relative to baseline. Overall F1 values are means, while attack-specific recall values are mean ± standard deviation.

Metric	Attack Configuration	Attack Strength	1.00	1.25	1.50
Overall F1	All evaluated attacks	0.25	0.645	0.602	0.564
Overall F1	All evaluated attacks	0.50	0.794	0.764	0.747
Overall F1	All evaluated attacks	0.75	0.874	0.846	0.831
Overall F1	All evaluated attacks	1.00	0.928	0.901	0.886
Recall	DDoS	0.25	0.870±0.039	0.788±0.059	0.719±0.051
Recall	Exfiltration	0.25	0.027±0.004	0.023±0.004	0.027±0.005
Recall	Reconnaissance	0.25	0.562±0.093	0.509±0.086	0.458±0.038

**Table 10:** Representative analytics-side computational footprint for seed 42. Serialized sizes exclude scalers and validation-score arrays. Latency measurements use 1 inference thread, 3 vectorized batches of 1000 observations per repetition, 10 warm-up repetitions, and 100 timed repetitions. Reported latency is the median normalized per 1000 observations.

Detector	Serialized Detector State	Median Inference Time per 1000 Observations
Isolation Forest	3582.0 KB	10.36 ms
One-Class SVM	59.7 KB	4.81 ms
Autoencoder	15.5 KB	0.29 ms
Max-Z baseline	0 KB	0.13 ms

**Table 11:** Illustrative high-score LITH hunt lead generated from the seed-42 DDoS scenario.

Lead Field	Experiment-supported Content
Device context	Environmental-sensor profile with low-rate normal behavior.
Calibrated anomaly priority	Empirical validation percentile = 1.000; raw Isolation Forest score = 0.7593 and is retained as a within-class tie-breaker; decision threshold = 0.990.
Changed indicators	UDP ratio 0.9048 (19 UDP packets out of 21 total packets), compared with a benign sensor ratio near 0.08; 4 unique destinations versus a normal value near 1; packet-size standard deviation 111.2 bytes versus approximately 28 bytes; and mean packet size 280.9 bytes versus approximately 147 bytes.
Observed context	Strong protocol-mix, destination, packet-size variability, and packet-size deviations consistent with the injected DDoS configuration; the observation contains 21 packets compared with a benign sensor center near 7.
Recommended analyst checks	Inspect destinations and flow rate; correlate contemporaneous alerts; verify authorization; isolate the device only if compromise is confirmed.
Automated attribution/containment	None. LITH ranks the lead but does not autonomously attribute the activity to a specific malware family or initiate containment.

**Table 12:** Boundary-case LITH hunt leads from seed 42. Changed indicators are the four largest absolute device-class-standardized deviations and are contextual indicators rather than model-based feature attributions.

Case	Device Class	Percentile	Raw IF Score	Changed Indicators
Borderline exfiltration	Smart camera	0.9904	0.5602	5 unique destinations (z=6.66); CPU utilization 48.1% (z=3.75); 1204 packets (z=2.18); process count 20 (z=1.01).
Benign false positive	Environmental sensor	0.9901	0.5780	3 unique destinations (z=5.57); packet-size standard deviation 49.8 bytes (z=2.15); CPU utilization 2.0% (z=−1.81); UDP ratio 0.125 (z=0.68).

**Table 13:** Isolation Forest hunt-lead ranking performance across 10 Monte Carlo runs. Queue budgets are normalized per 10,000 observations and evaluated within device-class-specific queues before pooling counts across device classes for each seed.

Lead Budget per 10,000 Observations	Precision@k	Recall@k
50	1.0000±0.0000	0.0150±0.0000
100	1.0000±0.0000	0.0300±0.0000
500	0.9993±0.0011	0.1499±0.0002

**Table 14:** Generator-consistency fidelity of the four changed indicators reported for true-positive Isolation Forest hunt leads at the baseline operating point.

Attack Configuration	True-positive Leads	Mean Indicator Fidelity	All Four Indicators Generator-consistent
DDoS	16,669	0.9995	99.81%
Exfiltration	10,926	1.0000	100.00%
Reconnaissance	16,627	0.9866	94.62%
Overall	44,222	0.9948	97.91%

**Table 15:** Limited external validation on CICIoT2023 using three semantically aligned network features. Values for Isolation Forest are mean ± standard deviation across seeds 42–51. Max-Z is deterministic under the fixed split.

Detector	Precision	Recall	F1	ROC-AUC	AP	FPR	PPV (1%)	PPV (0.1%)
Isolation Forest	0.175±0.033	0.00036±0.00009	0.00072±0.00018	0.625±0.027	0.878±0.007	0.00955±0.00030	0.00038	0.000038
Max-Z	0.9973	0.7017	0.8238	0.8353	0.9709	0.01075	0.3973	0.0613

## Data Availability

The synthetic data, experimental code, and supporting result files generated in this study are openly available in the LITH GitHub repository at https://github.com/Dr-Fbamashmoos/LITH-IoT-Threat-Hunt-Lead (accessed on 10 October 2026). Additional implementation details and analyses are provided in the Supplementary Materials. The external evaluation uses a public subsampled release derived from the CICIoT2023 dataset [29].

## Supplementary Materials

The following supporting information can be downloaded at: https://www.mdpi.com/article/10.3390/s26206618/s1, File S1: Detailed experimental methods, reproducibility protocol, statistical comparisons, sensitivity analyses, hunt-lead evaluation, analytics-side computational measurements, and limited external validation using CICIoT2023.

## Abbreviations

The following abbreviations are used in this manuscript:

AE	Autoencoder
AP	Average Precision
CI	Confidence Interval
CPU	Central Processing Unit
DDoS	Distributed Denial of Service
DoS	Denial of Service
FPR	False-Positive Rate
IDS	Intrusion Detection System
IF	Isolation Forest
IIoT	Industrial Internet of Things
IoMT	Internet of Medical Things
IoT	Internet of Things
IP	Internet Protocol
LIME	Local Interpretable Model-Agnostic Explanations
LITH	Lightweight IoT Threat Hunter
LSTM	Long Short-Term Memory
MQTT	Message Queuing Telemetry Transport
MSE	Mean Squared Error
OCSVM	One-Class Support Vector Machine
PPV	Positive Predictive Value
RAM	Random-Access Memory
RBF	Radial Basis Function
ReLU	Rectified Linear Unit
ROC-AUC	Area Under the Receiver Operating Characteristic Curve
RTP	Real-Time Transport Protocol
SHAP	SHapley Additive exPlanations
SIEM	Security Information and Event Management
SIGFRID	Scene Interaction Graphs for Interference Detection
SOC	Security Operations Center
SVM	Support Vector Machine
TCP	Transmission Control Protocol
TPR	True-Positive Rate
UDP	User Datagram Protocol
