# Supplementary material for: LITH: A Lightweight Behavioral Anomaly-to-Hunt-Lead Framework for IoT Sensor Ecosystems
Source: Sensors (Basel). 2026 Oct 19;26(20):6618. doi: 10.3390/s26206618 (PMC13708889; doi:10.3390/s26206618)
Supplement: Supplementary file 1 [file sensors-26-06618-s001.zip › sensors-4557402-File S1 Manuscript-Supplementary.pdf]

Supplementary Materials

LITH: A Lightweight Behavioral Anomaly-to-Hunt-Lead Framework for IoT Sensor Ecosystems

Fatmah Bamashmoos
Enas Khairullah
Faculty of Computing & Information Technology
King Abdulaziz University, Jeddah, Saudi Arabia

S1. Overview

These supplementary materials document the evaluation of the Lightweight IoT Threat Hunter (LITH), a behavioral anomaly-to-hunt-lead framework for Internet of Things (IoT) sensor ecosystems.

The primary evaluation is a controlled, reproducible synthetic telemetry benchmark. It includes benign-only detector fitting, temporal partitioning, empirical-percentile calibration, matched-seed statistical comparisons, feature ablation, sensitivity analyses, hunt-lead ranking, indicator-consistency evaluation, and analytics-side computational measurements.

A limited external network-feature stress test using the public CICIoT2023 dataset is also included. This external evaluation uses three semantically aligned network features rather than the full eight-feature LITH configuration.

The supplementary materials provide additional methodological details and numerical results that support the corresponding manuscript.

The experiments demonstrate feasibility under the reported conditions. They do not establish performance on physical resource-constrained endpoints or production IoT networks.

S2. Reproducibility Protocol

S2.1. Controlled Benchmark

The controlled experiment was repeated across 10 independent random seeds:

$$\mathcal{S} = \{42, 43, 44, 45, 46, 47, 48, 49, 50, 51\}.$$

Each run contains 50,000 benign observations and 5000 attack observations.

The benign observations are distributed among three device-class profiles:

- Environmental sensor: 16,667 observations.
- Smart camera: 16,667 observations.
- Actuator: 16,666 observations.

The attack-only evaluation set contains:

- Distributed denial-of-service (DDoS): 1667 observations.
- Data exfiltration: 1667 observations.
- Reconnaissance: 1666 observations.

Benign observations are divided temporally into training, validation, and test partitions using a 60%/20%/20% split.

Attack observations are reserved exclusively for testing. No attack observations or attack labels are used during feature scaling, model fitting, autoencoder early stopping, or anomaly-score calibration.

S2.2. Device-Class-Specific Fitting

The preprocessing and detector-fitting procedures are performed separately for each device class.

A `StandardScaler` is fitted using benign training observations only. For the main comparison, 2000 benign training observations are sampled without replacement per device class for each learned detector.

The autoencoder uses a separate disjoint holdout of 1500 benign observations per device class from the training partition for early stopping. This holdout does not overlap with the observations used for model fitting.

The benign validation partition is reserved exclusively for anomaly-score calibration.

S3. Behavioral Telemetry

LITH uses an eight-dimensional behavioral representation. Five features are network-derived, while three are optional host-level indicators.

Table S1: Behavioral telemetry features used in the controlled LITH benchmark.

No.	Feature	Source	Definition
1	Packet count	Network	Integer number of packets per observation.
2	Mean packet size	Network	Average packet size in bytes.
3	Packet-size standard deviation	Network	Standard deviation of packet size in bytes.
4	Unique destination IP count	Network	Number of distinct destination addresses.
5	UDP ratio	Network	UDP packets divided by total packets.
6	CPU utilization	System	Host CPU utilization percentage.
7	Memory utilization	System	Host memory utilization percentage.
8	Process count	System	Integer number of active processes.

The network-only configuration uses Features 1–5. The system-only configuration uses Features 6–8. The combined configuration uses all eight features.

S3.1. Physical-Consistency Constraints

Packet count, UDP packet count, unique-destination count, and process count are integer-valued.

The UDP ratio is calculated as:

$$r_{\text{UDP}} = \frac{N_{\text{UDP}}}{N_{\text{packets}}}.$$

The following constraints are enforced:

$$0 \leq N_{\text{UDP}} \leq N_{\text{packets}}$$

and

$$N_{\text{destinations}} \leq N_{\text{packets}}.$$

For example, the illustrative seed-42 high-score DDoS observation contains 19 UDP packets out of 21 total packets:

$$r_{\mathrm{UDP}} = \frac{19}{21} = 0.9048.$$

These constraints ensure packet-level numerical consistency within the feature generator. They do not establish that the observations originate from real packet captures.

S4. Temporal Drift Configuration

A sinusoidal drift with baseline amplitude 2.5% is applied to selected benign variables.

Each benign device-class sequence completes one full cycle over its complete generated sequence. Consequently, the 60%/20%/20% temporal partitions cover consecutive portions of that cycle.

Table S2: Benign device-class drift phase offsets.

Device class	Phase offset (cycles)
Environmental sensor	0.00
Smart camera	0.17
Actuator	0.34

Attack observations are independently generated from benign-like bases before attack perturbations are applied.

Table S3: Attack-generation drift phase offsets.

Attack configuration	Phase offset (cycles)
DDoS	0.15
Exfiltration	0.25
Reconnaissance	0.35

Each attack set spans its own complete sinusoidal cycle. The phase offsets indicate starting positions and do not make attack samples temporally aligned with the benign test partition.

S5. Detector Configurations

S5.1. Isolation Forest

Isolation Forest uses 100 estimators, a subsample size of 256, all input features, and the implementation's automatic contamination setting.

For the implementation, the continuous raw anomaly score is defined as:

$$r_{\mathrm{IF}}(x) = -\texttt{score\_samples}(x).$$

The sign inversion ensures that larger values indicate greater anomaly evidence.

S5.2. One-Class Support Vector Machine

The one-class support vector machine (OCSVM) uses a radial basis function kernel with:

$$\nu = 0.1, \qquad \gamma = \texttt{scale}.$$

The fitting tolerance is 0.001, and the kernel cache is 1000 MB.

Its raw anomaly score is:

$$r_{\mathrm{OCSVM}}(x) = -\texttt{decision\_function}(x).$$

S5.3. Shallow Autoencoder

The autoencoder follows a symmetric fully connected architecture:

$$8 \to 6 \to 4 \to 3 \to 4 \to 6 \to 8.$$

The hidden layers use rectified linear unit (ReLU) activations, while the output layer is linear.

Training uses Adam with learning rate 0.001, mean squared error loss, batch size 256, a maximum of 25 epochs, and early-stopping patience of four epochs.

For standardized input $\mathbf{z}$ and reconstruction $\hat{\mathbf{z}}$, the anomaly score is:

$$r_{\mathrm{AE}}(\mathbf{z}) = \frac{1}{d}\sum_{i=1}^{d}(z_i - \hat{z}_i)^2.$$

Across the 30 autoencoder fits (three device classes and 10 random seeds), every fit reached the maximum 25 epochs. The early-stopping condition was not triggered before that limit.

S5.4. Max-Z Comparator

The Max-Z baseline is defined as:

$$r_{\mathrm{Max\text{-}Z}}(\mathbf{z}) = \max_i |z_i|.$$

Here, z_i is the corresponding feature value standardized using the benign training profile of its device class.

Max-Z contains no fitted detector state beyond the standardization parameters.

S6. Calibration and Decision Rules

Each raw anomaly score is converted to an empirical percentile relative to the benign validation distribution of its detector and device class.

Let s denote the test anomaly score and let $s_1^{(v)}, \ldots, s_{n_v}^{(v)}$ denote the benign validation scores.

The empirical percentile is:

$$p(s) = \frac{1}{n_v}\sum_{j=1}^{n_v}\mathbb{I}(s_j^{(v)} \le s).$$

Validation observations tied with the test score are included by the less-than-or-equal relation.

The baseline decision rule is:

$$p(s) > 0.99.$$

This percentile measures the relative position of the score against benign calibration behavior. It is not a probability of compromise.

For hunt-lead ordering, the calibrated percentile is the primary priority key. The raw anomaly score is used as a secondary key only within the same detector and device class.

Raw detector scores are not directly compared between different device classes.

S7. Controlled Detection Results

Table S4: Overall detector performance across 10 Monte Carlo runs (mean ± standard deviation).

Detector	Precision	Recall	F1	ROC-AUC	AP	FPR
Isolation Forest	0.977 ± 0.002	0.884 ± 0.008	0.928 ± 0.004	0.989 ± 0.001	0.983 ± 0.002	0.0103 ± 0.0012
OCSVM	0.976 ± 0.002	0.906 ± 0.008	0.940 ± 0.004	0.987 ± 0.001	0.982 ± 0.002	0.0112 ± 0.0009
Autoencoder	0.976 ± 0.002	0.896 ± 0.020	0.934 ± 0.011	0.986 ± 0.004	0.981 ± 0.004	0.0110 ± 0.0010
Max-Z	0.974 ± 0.004	0.853 ± 0.008	0.909 ± 0.004	0.980 ± 0.001	0.966 ± 0.002	0.0113 ± 0.0017

Precision, recall, F1, and false-positive rate (FPR) are reported at the 99th-percentile operating point.

The area under the receiver operating characteristic curve (ROC-AUC) and average precision (AP) are derived from continuous calibrated ranking scores.

S7.1. Prevalence-Adjusted Precision

The controlled test set has an attack prevalence of approximately 33.3%.

To illustrate lower-prevalence conditions, expected positive predictive value (PPV) is calculated as:

$$\mathrm{PPV}(\pi) = \frac{\mathrm{TPR}\pi}{\mathrm{TPR}\pi + \mathrm{FPR}(1-\pi)}.$$

Here, π is attack prevalence, and TPR denotes true-positive rate or recall.

Table S5: Illustrative prevalence-adjusted positive predictive values in the controlled benchmark.

Detector	PPV at 1%	PPV at 0.1%
Isolation Forest	0.464	0.079
OCSVM	0.450	0.075
Autoencoder	0.452	0.075
Max-Z	0.433	0.070

These values are mathematical prevalence-adjusted estimates, not measurements from low-prevalence production networks.

S8. Statistical Comparisons

The same 10 Monte Carlo seeds are used in matched-detector comparisons.

Three planned paired t-tests compare OCSVM F1-score against Isolation Forest, the autoencoder, and Max-Z. Holm correction controls family-wise multiplicity.

Table S6: Paired F1-score comparisons across matched Monte Carlo seeds.

Comparison	Mean difference	95% CI	Holm-adjusted p	Cohen's d_z
OCSVM vs. IF	0.0111	0.0067 to 0.0155	5.49×10^{-4}	1.82
OCSVM vs. AE	0.0051	−0.0041 to 0.0144	0.240	0.40
OCSVM vs. Max-Z	0.0301	0.0257 to 0.0346	2.66×10^{-7}	4.88

The difference between OCSVM and the autoencoder was not statistically significant after Holm correction.

S8.1. Normality Diagnostics

Table S7: Shapiro–Wilk normality tests applied to paired F1-score differences.

Comparison	p-value
OCSVM vs. Isolation Forest	0.223
OCSVM vs. Autoencoder	0.061
OCSVM vs. Max-Z	0.281

S8.2. Nonparametric Robustness Check

Two-sided Wilcoxon signed-rank tests were also conducted using the matched seeds.

Table S8: Holm-adjusted Wilcoxon signed-rank test results.

Comparison	Holm-adjusted p-value
OCSVM vs. Isolation Forest	0.0059
OCSVM vs. Autoencoder	0.4316
OCSVM vs. Max-Z	0.0059

The Wilcoxon analysis supports the same qualitative conclusions as the paired t-tests.

S9. Attack-Specific Evaluation

Table S9: Attack-specific Isolation Forest performance across 10 Monte Carlo runs.

Attack	Precision	Recall	F1	ROC-AUC	AP
DDoS	0.942 ± 0.006	1.000 ± 0.000	0.970 ± 0.003	1.000 ± 0.000	0.998 ± 0.002
Exfiltration	0.914 ± 0.008	0.655 ± 0.024	0.763 ± 0.016	0.968 ± 0.003	0.882 ± 0.011
Reconnaissance	0.941 ± 0.006	0.998 ± 0.002	0.969 ± 0.003	1.000 ± 0.000	0.996 ± 0.002

The observed differences are specific to the evaluated attack-device configurations.

DDoS was applied to the environmental-sensor profile, exfiltration to the smart-camera profile, and reconnaissance to the actuator profile.

Consequently, attack type is confounded with device class and injected perturbation magnitude. These results should not be interpreted as an intrinsic ranking of attack difficulty.

S10. Training-Size and Hyperparameter Sensitivity

Table S10: Sensitivity to the number of benign fitting observations.

Detector	Baseline F1	Full-training F1	Mean paired difference	95% CI
Isolation Forest	0.9285	0.9251	−0.0034	−0.0090 to 0.0022
OCSVM	0.9396	0.9390	−0.0006	−0.0029 to 0.0018
Autoencoder	0.9344	0.9210	−0.0134	−0.0253 to −0.0016

The fixed 2000-observation fitting budget does not appear to artificially advantage Isolation Forest or OCSVM in this benchmark.

The autoencoder exhibits greater sensitivity to the larger fitting set under its unchanged architecture and training schedule.

Table S11: Isolation Forest tree-count sensitivity.

Trees	Mean F1	Mean ROC-AUC
50	0.9204 ± 0.0088	0.9873
100	0.9285 ± 0.0040	0.9891
200	0.9319 ± 0.0046	—

The unreported 200-tree ROC-AUC summary is shown as not available rather than estimated.

Table S12: Autoencoder bottleneck-width sensitivity.

Bottleneck width	Mean F1
2	0.9376 ± 0.0070
3	0.9344 ± 0.0109
4	0.9406 ± 0.0034

These local sensitivity analyses were performed after specifying the baseline configurations. They were not used to reselect the final hyperparameters from attack-labeled test results.

S11. Operating-Point Sensitivity

The anomaly-score calibration threshold is an operational policy choice rather than a universally optimal threshold.

Table S13: Isolation Forest operating-point sensitivity across 10 Monte Carlo runs.

Percentile	Precision	Recall	F1	FPR	Exfil. recall	Benign leads/10k	PPV 1%	PPV 0.1%
95.0	0.900	0.952	0.926	0.0527	0.857	527	0.154	0.018
97.5	0.946	0.925	0.935	0.0263	0.775	263	0.262	0.034
99.0	0.977	0.884	0.928	0.0103	0.655	103	0.464	0.079
99.5	0.987	0.854	0.915	0.00567	0.567	57	0.603	0.131

Lowering the operating threshold improves exfiltration recall but increases the number of benign investigation leads.

Lead counts are normalized per 10,000 benign observations rather than expressed per day because the feature-level benchmark does not specify a physical observation cadence.

S12. Feature-Ablation Analysis

Table S14: Isolation Forest feature-ablation results across 10 Monte Carlo runs.

Feature set	Precision	Recall	F1	ROC-AUC
Network-only	0.977 ± 0.003	0.886 ± 0.014	0.929 ± 0.009	0.989 ± 0.001
System-only	0.896 ± 0.031	0.206 ± 0.037	0.334 ± 0.050	0.810 ± 0.007
Combined	0.977 ± 0.002	0.884 ± 0.008	0.928 ± 0.004	0.989 ± 0.001

The paired network-only versus combined mean F1 difference is 0.0007, with a 95% confidence interval from −0.0072 to 0.0087.

The paired t-test gives $p = 0.843$, with Cohen's $d_z = 0.065$.

This comparison does not establish that adding system telemetry reduces performance. Instead, network-only features were sufficient to retain nearly the same performance for the three simulated attack configurations.

S13. Attack-Strength and Benign-Variability Analysis

Attack strength was varied across:

$$a \in \{0.25, 0.50, 0.75, 1.00\}.$$

Benign variability multipliers were:

$$v \in \{1.00, 1.25, 1.50\}.$$

Table S15: Mean overall Isolation Forest F1-score across attack-strength and benign-variability conditions.

Attack strength	Variability 1.00	Variability 1.25	Variability 1.50
0.25	0.645	0.602	0.564
0.50	0.794	0.764	0.747
0.75	0.874	0.846	0.831
1.00	0.928	0.901	0.886

Performance decreases as the injected attack becomes weaker and benign behavior becomes more variable.

Table S16: Attack-specific recall at quarter-strength attacks ($a = 0.25$), mean ± standard deviation.

Attack	Variability 1.00	Variability 1.25	Variability 1.50
DDoS	0.870 ± 0.039	0.788 ± 0.059	0.719 ± 0.051
Exfiltration	0.027 ± 0.004	0.023 ± 0.004	0.027 ± 0.005
Reconnaissance	0.562 ± 0.093	0.509 ± 0.086	0.458 ± 0.038

Table S17: Mean exfiltration recall across attack strengths and benign-variability levels.

Attack strength	Variability 1.00	Variability 1.25	Variability 1.50
0.25	0.0268	0.0232	0.0275
0.50	0.1323	0.0957	0.0968
0.75	0.4004	0.3043	0.2801
1.00	0.6549	0.5147	0.4533

Weak exfiltration behavior remains particularly difficult to detect at the fixed baseline threshold.

These sensitivity experiments are controlled feature-level perturbations; they do not reproduce every property of naturally occurring low-and-slow attacks.

S14. Hunt-Lead Ranking

LITH converts anomaly scores into investigation leads.

Ranking is performed separately within each device-class queue. The calibrated empirical percentile serves as the primary key, while the raw score is used as a secondary ordering key within a detector and device class.

The evaluation uses normalized queue budgets of 50, 100, and 500 leads per 10,000 observations.

Table S18: Isolation Forest hunt-lead ranking performance across 10 Monte Carlo runs.

Lead budget/10k	Precision@k	Recall@k
50	1.0000 ± 0.0000	0.0150 ± 0.0000
100	1.0000 ± 0.0000	0.0300 ± 0.0000
500	0.9993 ± 0.0011	0.1499 ± 0.0002

These results are specific to the synthetic benchmark and its high attack prevalence. They should not be used as estimates of production investigation-queue precision.

S15. Changed-Indicator Fidelity

Each LITH hunt lead includes four contextual indicators selected as the features with the greatest absolute device-class-standardized deviations:

$$\mathrm{TopFour}(|z_i|).$$

These indicators are not model-based causal explanations.

For true-positive synthetic leads, generator-consistency fidelity measures the fraction of reported indicators belonging to features intentionally modified by the corresponding attack generator.

Table S19: Changed-indicator generator-consistency fidelity for true-positive Isolation Forest hunt leads.

Attack	True-positive leads	Mean fidelity	All four consistent
DDoS	16,669	0.9995	99.81%
Exfiltration	10,926	1.0000	100.00%
Reconnaissance	16,627	0.9866	94.62%
Overall	44,222	0.9948	97.91%

The perfect fidelity of the exfiltration configuration must be interpreted cautiously because its synthetic generator modifies all eight evaluated features.

This metric measures consistency with known perturbations, not causal explanation accuracy.

S16. Percentile Saturation and Case Examples

S16.1. Percentile Saturation

Multiple observations may share the maximum empirical percentile of 1.000.

Table S20: Seed-42 observations assigned the maximum empirical validation percentile of 1.000.

Observation category	Count at percentile 1.000
DDoS	1658 / 1667
Exfiltration	322 / 1667
Reconnaissance	1291 / 1666
Benign	4 / 10,002

The raw detector score is therefore retained as a secondary ordering criterion within the same detector and device class.

S16.2. Illustrative High-Score DDoS Lead

The selected seed-42 observation has raw Isolation Forest score approximately 0.7593 and calibrated percentile 1.000.

Its recorded feature values include:

- Packet count: 21.
- Mean packet size: 280.9 bytes.
- Packet-size standard deviation: 111.2 bytes.
- Unique destination count: 4.
- UDP ratio: $19/21 = 0.9048$.
- CPU utilization: 7.34%.
- Memory utilization: 24.15%.
- Process count: 11.

The leading standardized deviations are UDP ratio, unique destination count, packet-size standard deviation, and mean packet size.

The lead is provided for analyst investigation and does not constitute automated malware attribution.

S16.3. Boundary-Case Investigation Leads

A borderline exfiltration observation is selected as the detected exfiltration case with the smallest percentile above the baseline decision boundary.

Its percentile is 0.9904 and its raw Isolation Forest score is 0.5602.

The reported indicators are five unique destinations ($z = 6.66$), CPU utilization 48.1% ($z = 3.75$), 1204 packets ($z = 2.18$), and process count 20 ($z = 1.01$).

A benign false-positive observation is selected as the benign observation with a percentile closest above the 0.99 threshold.

Its percentile is 0.9901 and its raw score is 0.5780.

The indicators are three unique destinations ($z = 5.57$), packet-size standard deviation 49.8 bytes ($z = 2.15$), CPU utilization 2.0% ($z = -1.81$), and UDP ratio 0.125 ($z = 0.68$).

These examples demonstrate why threshold crossings must be treated as investigation leads rather than confirmed malicious activity.

S17. Analytics-Side Computational Footprint

The representative computational-footprint evaluation used the seed-42 detector bundle.

The evaluation environment exposed five Intel Xeon Platinum 8573C logical CPU cores on Linux x86_64 and approximately 5.81 GB of visible random-access memory.

The software versions were:

- Python 3.13.5.
- NumPy 2.3.5.
- pandas 2.2.3.
- SciPy 1.17.0.
- scikit-learn 1.8.0.
- PyTorch 2.10.0+cpu.

The latency benchmark used one inference thread, 10 untimed warm-up repetitions, and 100 timed repetitions.

Each timed repetition processed three vectorized batches of 1000 observations, one batch per device class.

The reported latency is the median wall-clock time normalized per 1000 observations.

The timed path includes feature standardization and raw anomaly scoring but excludes empirical-percentile lookup.

Table S21: Representative analytics-side detector footprint for seed 42.

Detector	Serialized detector state	Median time per 1000 observations
Isolation Forest	3582.0 KB	10.36 ms
OCSVM	59.7 KB	4.81 ms
Autoencoder	15.5 KB	0.29 ms
Max-Z	0 KB	0.13 ms

Serialized sizes exclude fitted scalers and validation-score calibration arrays.

These results characterize general-purpose analytics execution and must not be interpreted as measurements of endpoint collection overhead, embedded-edge inference, or device energy consumption.

S18. Limited External Validation on CICIoT2023

S18.1. Dataset and Scope

A limited external network-feature stress test was conducted using a public processed subset of CICIoT2023.

The original CICIoT2023 collection contains traffic from an IoT environment with 105 devices and represents 33 attack types grouped into seven attack families.

The evaluation used the public `random_3way` stratified subsampled release.

The processed representation does not provide direct counterparts for every LITH telemetry feature or the required device-class identifiers.

Therefore, the experiment was restricted to three semantically aligned network features:

- `Number`: packet count.

- `AVG`: average packet length.
- `Std`: packet-length standard deviation.

The dataset's protocol indicator was not treated as equivalent to LITH's UDP-packet ratio.

No destination-count feature was fabricated.

Accordingly, this evaluation is a limited network-feature stress test and not a full replication of the eight-feature, device-class-specific LITH framework.

S18.2. Split and Calibration Protocol

The public training split was restricted to benign observations for feature scaling and detector fitting.

The public test split was restricted to benign observations for empirical-percentile calibration.

The public validation split, containing benign and attack observations, was reserved for final evaluation.

Isolation Forest used 100 trees and 2000 benign fitting observations for each seed from 42 through 51.

Max-Z used the same standardized features and calibration protocol.

The decision rule was:

$$p(s) > 0.99.$$

No attack labels were used for fitting, scaling, or threshold calibration.

S18.3. External Results

Table S22: Limited CICIoT2023 three-feature external stress-test results. Isolation Forest values are mean ± standard deviation over 10 seeds; Max-Z is deterministic under the fixed split.

Detector	Precision	Recall	F1	ROC-AUC	AP	FPR	PPV 1%	PPV 0.1%
Isolation Forest	0.175 ± 0.033	0.00036 ± 0.00009	0.00072 ± 0.00018	0.625 ± 0.027	0.878 ± 0.007	0.00955 ± 0.00030	0.00038	0.000038
Max-Z	0.9973	0.7017	0.8238	0.8353	0.9709	0.01075	0.3973	0.0613

The external results show that the detector ranking observed in the controlled benchmark did not persist under the reduced three-feature representation.

In particular, Isolation Forest had extremely low recall at the fixed calibration operating point, despite nonzero continuous-score ranking performance.

Max-Z performed substantially better under the same external evaluation setting.

Because the external validation set is attack-heavy, the observed precision and average precision should not be interpreted as production performance under rare attack conditions.

S18.4. Attack-Family Dependence

Max-Z recall was approximately 1.000 for DDoS, denial-of-service (DoS), and Mirai traffic.

However, recall was approximately:

- 0.0020 for reconnaissance.
- 0.0003 for spoofing.
- 0.0035 for web-based attacks.
- 0.0054 for brute-force attacks.

These results indicate that the strong aggregate Max-Z performance is driven mainly by attack families producing large deviations in the retained traffic-volume and packet-size features.

The external results do not establish reliable cross-dataset generalization for all attack families.

S19. Interpretation and Limitations

The controlled benchmark enables reproducible comparisons under documented distributional assumptions.

However, the benign and malicious observations are generated within the same author-designed feature space.

The strong Max-Z baseline confirms that some controlled attack configurations contain substantial univariate feature shifts.

Although robustness analyses reduce concerns that the findings depend on a single favorable attack magnitude, they do not eliminate the limitations of feature-level simulation.

The external CICIoT2023 evaluation provides a separate test of limited network-feature transfer.

Its substantially different results demonstrate that the detector ranking and operating-point behavior observed synthetically are not universally transferable.

The external experiment does not reconstruct the full device-class-specific LITH system and does not measure the performance of the complete threat-hunting workflow.

Additional limitations include:

- No physical endpoint or embedded-edge resource evaluation.
- No raw packet-level implementation benchmark.
- No experimentally specified aggregation-window duration.
- Attack-device-profile confounding in the controlled benchmark.
- No production security operations center workflow validation.
- Limited detection of weak synthetic exfiltration at strict thresholds.
- No claim of autonomous threat confirmation or attribution.
- No evidence of reliable detection across all external attack families.

The present results support the feasibility of converting calibrated behavioral deviations into contextualized, ranked investigation leads.

They do not establish deployment readiness.

S20. Reproducibility Artifacts

The experimental code, synthetic dataset, model configurations, and machine-readable experimental results are publicly available through the LITH reproducibility repository:

`https://github.com/Dr-Fbamashmoos/LITH-IoT-Threat-Hunt-Lead`

The repository provides supporting artifacts for the controlled benchmark, statistical comparisons, sensitivity analyses, hunt-lead evaluation, and computational-footprint measurements.

The present supplementary document reports the additional methodological details and quantitative results, while the repository provides access to the corresponding computational artifacts for independent inspection and reproduction.

S21. Supplementary References

Neto, E.C.P.; Dadkhah, S.; Ferreira, R.; Zohourian, A.; Lu, R.; Ghorbani, A.A. CICIoT2023: A Real-Time Dataset and Benchmark for Large-Scale Attacks in IoT Environment. *Sensors* **2023**, *23*, 5941.
`https://doi.org/10.3390/s23135941`
